# Dissecting the Effect of Genetic Variation on the Hepatic Expression of Drug Disposition Genes across the Collaborative Cross Mouse Strains

**DOI:** 10.3389/fgene.2016.00172

**Published:** 2016-10-05

**Authors:** Aharon Nachshon, Hanifa J. Abu-Toamih Atamni, Yael Steuerman, Roa'a Sheikh-Hamed, Alexandra Dorman, Richard Mott, Juliane C. Dohm, Hans Lehrach, Marc Sultan, Ron Shamir, Sascha Sauer, Heinz Himmelbauer, Fuad A. Iraqi, Irit Gat-Viks

**Affiliations:** ^1^Department of Cell Research and Immunology, Faculty of Life Sciences, Tel-Aviv UniversityTel-Aviv, Israel; ^2^Department of Clinical Microbiology and Immunology, Sackler Faculty of Medicine, Tel- Aviv UniversityTel-Aviv, Israel; ^3^Genetics Institute, University College of LondonLondon, UK; ^4^Genomics Unit, Center for Genomic RegulationBarcelona, Spain; ^5^Universitat Pompeu FabraBarcelona, Spain; ^6^Department of Biotechnology, University of Natural Resources and Life Sciences Vienna (BOKU)Vienna, Austria; ^7^Department of Vertebrate Genomics, Max Planck Institute for Molecular GeneticsBerlin, Germany; ^8^The Blavatnik School of Computer Science, Tel Aviv UniversityTel Aviv, Israel; ^9^CU Systems Medicine, University of WürzburgWürzburg, Germany

**Keywords:** collaborative cross mouse strains, hepatic drug disposition, eQTLs analysis, transcript isoforms, genetic variation

## Abstract

A central challenge in pharmaceutical research is to investigate genetic variation in response to drugs. The Collaborative Cross (CC) mouse reference population is a promising model for pharmacogenomic studies because of its large amount of genetic variation, genetic reproducibility, and dense recombination sites. While the CC lines are phenotypically diverse, their genetic diversity in drug disposition processes, such as detoxification reactions, is still largely uncharacterized. Here we systematically measured RNA-sequencing expression profiles from livers of 29 CC lines under baseline conditions. We then leveraged a reference collection of metabolic biotransformation pathways to map potential relations between drugs and their underlying expression quantitative trait loci (eQTLs). By applying this approach on proximal eQTLs, including eQTLs acting on the overall expression of genes and on the expression of particular transcript isoforms, we were able to construct the organization of hepatic eQTL-drug connectivity across the CC population. The analysis revealed a substantial impact of genetic variation acting on drug biotransformation, allowed mapping of potential joint genetic effects in the context of individual drugs, and demonstrated crosstalk between drug metabolism and lipid metabolism. Our findings provide a resource for investigating drug disposition in the CC strains, and offer a new paradigm for integrating biotransformation reactions to corresponding variations in DNA sequences.

## Introduction

Drug disposition encompasses the processes of drug absorption into the bloodstream, drug metabolism into different chemicals (mainly in the liver and intestine), distribution of the various chemicals into different tissues, and removal of the chemicals from the body through excretion. The organism's genetic makeup might play a part in the activity of any of these processes and might underlie chemical toxicity and adverse drug reactions (Meyer, 2000). To address this problem, a key goal of predictive medicine is to identify the DNA loci, termed quantitative trait loci (QTLs), which can be used to predict the response to a given medication and its toxicity in a particular patient (e.g., Rost et al., 2004; Harrill and Rusyn, 2008). This challenge can be easily and systematically addressed by utilizing specific mouse models in preclinical pharmacogenetics research.

Mouse pharmacogenetic studies have typically been applied across F2 progeny and backcross populations (Rusyn et al., 2010; Frick et al., 2013); across recombinant inbred (RI) lines derived by crossing two founder strains and inbreeding during many generations (Cook et al., 2004; Hitzemann et al., 2004); and across a predefined collection of classical inbred lines (Montgomery et al., 2013; Yoo et al., 2015). Although these approaches have proved useful in many studies, they are derived mainly from *Mus musculus domesticus* and thus reflect only a partial repertoire of adverse effects, limiting pharmacogenetic investigation.

A promising new model organism has been provided by the recently developed Collaborative Cross (CC) strains, a large, genetically diverse mouse reference population. The CC panel is a collection of RI mouse lines that combine the genomes of eight genetically and phenotypically diverse founder strains—A/J, C57BL/6J [B6], 129S1/SvImJ, NOD/ShiLtJ, NZO/HlLtJ, CAST/EiJ, PWK/PhJ, and WSB/EiJ (Aylor et al., 2011; Consortium, 2012). Whereas the first five founders are classical *Mus musculus domesticus* subspecies, the last three are wild-derived strains representing the three *Mus musculus* subspecies, namely *M. m. musculus, M. m. domesticus*, and *Mus m. castaneus*. Thus, like classical RI strains, CC mice are genetically reproducible with balanced allele frequencies, but in addition they incorporate a large amount of genetic variation and dense recombination sites (Valdar et al., 2006; Roberts et al., 2007; Yang et al., 2011; Chesler, 2014). The availability of genotyping data across all CC lines opened the way to analyses of QTLs in a variety of traits, including behavioral and morphological phenotypes (Aylor et al., 2011; Chesler, 2014; Mao et al., 2015; Percival et al., 2016), homeostatic immune processes (Kelada et al., 2012; Phillippi et al., 2014), susceptibility to infectious diseases (Durrant et al., 2011; Ferris et al., 2013; Vered et al., 2014; Graham et al., 2015; Gralinski et al., 2015; Lorè et al., 2015), and liver-related functionalities (Kelada et al., 2012; Svenson et al., 2012; Thaisz et al., 2012).

While the CC population has been proven phenotypically diverse, the extent to which drug disposition varies across these strains is still largely unknown (Rusyn et al., 2010; Gelinas et al., 2011; Frick et al., 2013). One of the many mechanisms through which variation in drug disposition can arise is the biotransformation of drugs in the liver. In such biotransformation, drug metabolizing enzymes (DMEs) and drug transport proteins (DTPs) catalyze the biochemical modification and transport of exogenous chemicals and other xenobiotics (Katz et al., 2008). With regard to hepatic drug metabolism in the CC lines, two key questions arise: (i) Should a large diversity be expected in hepatic biotransformation of particular drugs? (ii) Can CC mice be used to evaluate the crosstalk between drug metabolism and other functionalities of the liver, especially those related to lipid and fatty acid metabolism?

Here we exploit transcriptional mechanisms to dissect genetic variation in hepatic drug metabolism of the CC lines. We focus on *cis*-regulatory variants underlying inter-individual variation in gene expression. Such genetic variants, referred to as “proximal eQTLs,” are central to the understanding of metabolic diversity owing to their relatively large genetic effect size (Wittkopp and Kalay, 2012; Bryois et al., 2014) and the plasticity of proximal factors over short evolutionary time scales (Wray, 2007). Proximal regulatory variation is known to affect metabolic traits (e.g., Hsieh et al., 2007; Zhong et al., 2010; Kang et al., 2012), but the organization and architecture of such elements in governing hepatic drug metabolism has not yet been subjected to comprehensive characterization.

We leverage RNA-sequencing technology (Mortazavi et al., 2008; Rosenkranz et al., 2008; Parkhomchuk et al., 2009) to generate transcription profiles of the liver tissues from 29 CC lines (55 individuals). By applying eQTL analysis on these data we characterized the genetic control over expression of drug disposition enzymes—either control of the overall expression of genes or of the expression of particular transcript isoforms. Using these predictions we compiled a network of connectivity between genomic loci and the metabolism of specific drugs, highlighting potential joint genetic effects and drug-drug interactions. Further analysis of nuclear receptors participating in the regulation of cellular-biochemical pathways provided DNA variants that affect the crosstalk between lipid metabolism and drug metabolism. Thus, our study supports the premise that the CC population is a valuable model system for the investigation of genetic variation in response to a wide range of chemicals and drugs, and may further offer mechanisms by which DNA variation contributes to the relationship between lipid metabolism and adverse clinical effects. Moreover, our approach provides a general strategy for a system-level mapping of eQTL-drug connectivity across a genetically diverse population.

## Materials and methods

### CC lines

The Collaborative Cross (CC) recombinant inbred mouse lines were used as described elsewhere (Iraqi et al., 2008; Durrant et al., 2011; Consortium, 2012). Animals were housed on hardwood chip bedding in open top cages at the animal facility of Tel-Aviv University (TAU) under a 12-h light/dark cycle. Mice were given tap water and rodent chow *ad libitum* throughout the experiment. Liver tissues were collected from 8- to 10-week old male CC mice from the TAU cohort at inbreeding generation between 16 and 42. A total of 55 mice from 29 CC lines were used (Table S1). All experimental protocols were approved by the Institutional Animal Care and Use Committee (IACUC) at TAU (approved protocol M-13-033) according to the national guidelines.

### RNA extraction, RNA-Seq library preparation, and sequencing

The liver tissues were dissected and subsequently stored in sterilized tubes at −196°C (in liquid nitrogen). The RNA was extracted using the RNeasy Plus kit procedure (Qiagen). RNA quality was tested on a 2100 BioAnalyzer (Agilent); in all samples, RNA Integrity number (RIN) exceeded 7. The RNA-Seq libraries were prepared using the TruSeq Stranded mRNA library preparation kit (Illumina). Libraries were pooled and sequenced on the Illumina HiSeq 2000 and 2500 sequencers with Illumina v3 sequencing chemistry. Paired-end sequencing was performed by reading 50 bases at each end of a fragment. Overall, each sample consisted of 24–37.5 M RNA-sequencing fragments with an average of 31.5 M fragments. This data is accessible through GEO Series accession number GSE77715. A detailed description of our data analysis appears below (see a summary of the computational pipeline in Table S2).

### RNA-Seq quantification

RNA-Seq data was mapped and quantified using RSEM version 1.2.18 (Li and Dewey, 2011) with the mouse genome (UCSC, mm9, NCBI37) and annotation file (Ensembl version 37.67). The reference was created by the RSEM rsem-prepare-reference command, followed by calculation of the expression level of genes using the rsem-calculate-expression command. The analysis was applied with default parameters and using Bowtie 2 version 2.1.0 (Langmead and Salzberg, 2012). Overall, the average percentage of unmapped fragments was 11.9%, (min = 9.4%, max = 24.3%); the average percentage of fragments aligned to a single gene was 59.9% (min = 49.6%, max = 69.5%), and among them, fragments aligned to just one isoform were 32.6% (min = 26.9%, max = 38.7%).

*Total expression levels* were measured by RSEM's FPKM metric, defined as the number of fragments mapped to the genomic region of a gene per kilobase of the gene's exons and per million mapped fragments. An *isoform-ratio level*, in contrast, is defined as the percentage of detectable fragments mapped to a given alternatively spliced isoform of a gene, out of the total number of fragments mapped to that gene (the RSEM's IsoPct metric). The *expression trait* of a gene refers to either the overall expression (a *total-expression trait*) or the percentage of a specific isoform (an *isoform-ratio trait)*. In the following we describe the transformation and association test steps, which are applied separately for each trait. For simplicity, we omit the index of the gene (either a total-expression trait or an isoform-ratio trait) whenever possible.

### Data transformation

For a given expression trait, denote its level in individual *i* by *v*_*i*_. The maximal level of a trait, denoted *v*_*M*_, is $\max{\left\{v_{i}\right\}}_{i=1}^{n}$, where *n* is the number of individuals. The *bin size* δ of a trait is defined as $\max\left\{0.5,\frac{v_{M}}{200}\right\}$, where the first term (0.5) represents an accuracy threshold and the second term represents 0.5% of the maximum level.

Let $v_{i}^{*}$ be the rounded level of *v*_*i*_ to the accuracy of bin size δ. Based on these rounded levels, the van der Waerden normal scores transformation (Lehmann, 1975; Aylor et al., 2011) is defined by

$$\begin{array}{l} u_{i}=\phi^{-1}\left(\frac{r_{i}^{*}}{n+1}\right), \end{array}$$

where *u*_*i*_ is the adjusted level of the trait in individual *i*, ϕ^−1^ (*p*) is the quantile function of the normal distribution with probability *p*, and $r_{i}^{*}$ is the rank of $v_{i}^{*}$ among the *n* values $\left\{v_{1}^{*},v_{2}^{*},\ldots ,v_{n}^{*}\right\}$ with ties resolved by the average rank. Throughout this study, we refer to the adjusted traits ${\left\{u_{i}\right\}}_{i=1}^{n}$ rather than to the original measurements.

### Association tests

An association score between a given expression trait and a given genome interval was calculated by regressing the trait on the contribution of their eight CC founder strains as previously described (Mott et al., 2000). More formally, for a given genome interval and a trait, the *association score* is the likelihood ratio (LR) between the null model *u*_*i*_ = μ + β*_m_i__* + ε_*i*_ and the genetic model:

(1) $$\begin{array}{l} u_{i}=\mu +\overset{8}{\underset{k=1}{\sum }}\alpha_{k}g_{k,m_{i}}+\beta_{m_{i}}+\varepsilon_{i}, \end{array}$$

where *m*_*i*_ is the CC line of the *i*-th individual; *g*_*k*,_*m*__*i*__ is the haplotype probability of the *k*-th founder (*k* ∈ {1, …, 8}) in CC line *m*_*i*_, μ is the intercept value, α_*k*_ is the genetic fixed effect of the *k*-th founder, β_*m*_ is the random effect of CC line *m*, and an error term ε_*i*_ is assumed to be normally distributed ε ~ *N*(0, σ^2^). No other covariates were used. The mixed model regression was implemented in R by using the *lme4* package version 1.1-7 (Bates et al., 2015).

The false discovery rate (FDR) was estimated by comparing the LR values in real data to the distribution of the LR values using randomly permuted datasets. Throughout this study, the *permutation FDR* was calculated as *C*_*perm*_/*C*_*real*_ where *C*_*real*_ and *C*_*perm*_ denote the number of traits with LR values exceeding a certain threshold in the original (real) dataset and in the permuted dataset, respectively (*C*_*perm*_ is averaged over 100 permuted datasets). In order that the permutations will specifically randomize the fixed effect (the genetics) rather than the random effect, the shuffling was applied on the CC line labels.

We applied the association tests to genes that are expressed in the liver (*v*_*M*_ ≥ 2) and to a single isoform-ratio traits from each of these genes: the isoform with the highest number of different rounded levels in the set ${\left\{v_{i}^{*}\right\}}_{i=1}^{n}$. Since we are interested in the variation in expression and to reduce the running time of the analysis, we further excluded traits with a low (< 12) number of different rounded levels across the 55 individuals. Altogether, the association testes were applied on 5950 total-expression traits and 4712 isoform-ratio traits.

The association tests were applied on genotyping dataset that was obtained from the UNC systems genetics repository (http://csbio.unc.edu/CCstatus) and included measurements from MegaMUGA—a 77K marker genotyping array based on the Illumina Infinium platform. The genotyping data was computationally validated using comparison with the polymorphic loci in the RNA-Seq data (Figure S1). For a given genome interval, the haplotype probabilities *g*_*k, m*_ of each of founder *k* in each CC line *m* were calculated using the HAPPY package version 2.4 (Mott et al., 2000). Altogether, the association tests were applied on 23,217 genome intervals for which the haplotype probabilities were calculated. Unless stated otherwise, the analysis was focused on proximal genome intervals. To that end, association scores were calculated using genomic intervals whose distance to the gene's transcription start site is less than 5 Mbp.

Throughout the manuscript, we use the following terminology: a *proximal eQTL* is defined as a nearby genome interval whose FDR is lower than 0.01 (based on 100 permutations). There are two types of proximal eQTLs: *total-expression eQTLs* that are associated with the expression level of total-expression traits, and *isoform-ratio eQTLs* that are associated with the percentage of alternatively spliced isoforms (see Tables S3, S4 for full lists).

We note that it is possible to use the optimized regression parameters (from equation 1) to determine the contribution of each founder to the overall regulatory variation. As previously described (Aylor et al., 2011; Durrant et al., 2011), we define the *founder effect* as $\frac{\alpha_{k}}{\max{\left\{abs\left(\alpha_{k}\right)\right\}}_{k=1}^{8}}$: the higher the (absolute) founder effect on a certain eQTL target, the stronger the contribution of the DNA changes in the eQTL region of this founder strain.

### Construction of an eQTL-drug connectivity map

The eQTL-drug connectivity map was generated in several steps. In step 1 we assembled a reference collection of manually curated drug-specific sets of enzymes. Each set in this collection includes a group of genes that play a role in the metabolic reactions of a particular drug, based on direct experimental evidence. The reference collection consists of 881 gene sets relating to 165 different drugs, which were assembled from the Kyoto Encyclopedia of Genes and Genomes (KEGG) and from the IPA database (QIAGEN, Redwood City, CA). In step 2, additional enzymes were added to each set based on indirect evidence. Specifically, each of the manually curated sets was further expanded with the alternative genes of the same chemical reaction (that is, with the same EC numbers). Altogether, steps 1 and 2 produced a reference collection of drug-specific gene sets, where the assignment of a gene to a particular set is based on either direct (step 1) or indirect (step 2) experimental evidence. Next, in step 3 we removed genes that were not associated with a proximal eQTL. In particular, given the reference collection from step 2, we retained only those genes that were significantly associated with at least one proximal eQTL (using the same thresholds as detailed above; see final collection in Tables S5, S6).

Building on this collection, the eQTL-drug connectivity map consists of three types of nodes: drugs with at least one non-empty curated gene set, genes in these non-empty sets, and the proximal eQTLs of these genes. The map contains edges between each drug node and its corresponding nodes of genes, as well as edges between each gene node and the nodes of its proximal eQTLs.

### Demonstration of genetic variation in splicing events

To demonstrate the alternative splicing events of a particular gene in a given sample, reads were aligned to the genome using Tophat version 2.0.9 (Kim et al., 2013) with –max-intron-length 20,000 (thus avoiding most cases of aligning the same fragment to two nearby genes). Uniquely mapped reads were extracted by filtering out those reads carrying poor mapping quality (< 10). Using the TopHat alignment, the IGV software (Thorvaldsdóttir et al., 2013) was used to show the reads' coverage together with the amount of splice junctions (see details below).

## Results

### Characterization of proximal eQTLs acting on hepatic drug disposition enzymes

To investigate transcription diversity in the livers of CC mice, we sequenced total RNA from the livers of 29 distinct CC lines (1–3 individuals per strain, 55 individuals in total; Table S1). On the basis of these data we quantified the total expression of each gene (“total-expression traits”) as well as the relative expression of the annotated isoforms (“isoform-ratio traits”; see Materials and Methods and Table S2). We found that the global expression profiles are similar between individuals of the same strains for both total-expression and isoform-ratio traits (Figures S2, S3); this dataset is therefore amenable to our study. In the following we focus on 5950 total expression traits and 4712 isoform-ratio traits that were highly variable across the CC mice.

We tested the association of each trait separately against all polymorphisms. A genome-wide map indicates, as expected, that this analysis has no spurious *trans*-eQTL bands (Figure S4) and do not show inflation of the association test statistics (Figure S5). The analysis is focused on proximal eQTLs, using a cutoff of 5 Mbp as evidence for *cis* regulatory variation. This permissive genomic range was selected to account for the lack of precision in the associated genome intervals (Figure S6). We used permutation to establish the null distribution of the association test statistics and then exploited the null distribution to calculate a permutation-based false discovery rate (“permutation FDR”) score (see Materials and Methods). At a permutation FDR of 0.01 we identified proximal eQTLs associated with the total expression of 365 genes (“total-expression eQTLs”) and associated with the expression of 243 specific transcript isoforms (“isoform-ratio eQTLs”), a total of 608 significantly associated traits (see Materials and Methods and Tables S3, S4). We note that the similar numbers of total-expression eQTLs and isoform-ratio eQTLs is in accordance with previous studies in human cohorts (Gonzàlez-Porta et al., 2012; Battle et al., 2014). Of the 3400 genes found to have dual annotation (both total-expression and isoform-ratio traits), in 43 we obtained both total-expression eQTL and isoform-ratio eQTL, not necessarily in the same genomic interval.

To characterize biochemical networks in the context of inherited transcriptional variation, we examined the 608 eQTL-associated traits and found them to be enriched in a curated list of biotransformation reactions from the “Ingenuity Pathway Analysis” (IPA) database (*P* < 0.03, hyper-geometric test). Specifically, 40 drug disposition genes were found to be associated with a proximal eQTL, which could be further classified according to their particular type of enzymatic reaction (Table 1):

- Functionalization reactions by oxidation, reduction and hydrolysis, which either activate or detoxify the drug. Among the eQTL-associated functionalization DMEs, oxidation is catalyzed by cytochrome P450 (*Cyp2a/2c/2d/3a*), alcohol and aldehyde dehydrogenases (*Adhfe1* and *Aldh8a1/16a1*), thiol-disulfide oxidoreductase (*Glrx2*), and FMO (*Fmo1*); reduction is catalyzed by aldo-keto reductases (*Akr1c13/19*); and hydrolysis is catalyzed by various esterases (*Ces1g/2h/3a, Siae, Sulf2*), epoxide hydrolase (*Ephx2*), dihydropyrimidinase (*Dpys*), glucuronidase (*Gusb*), and glyoxalase (*Glo1*).
- Conjugation reactions that transfer a functional group from a cofactor to a substrate chemical, resulting in detoxification followed by excretion. The eQTL-associated conjugation DMEs catalyze the transfer of various functional groups, including UDP-glucuronosyl, amino acid, N-acetyl, methyl and glutathione-S (*Ugt1a/Ugt3a, Ccbl1/2, Nat8, Tpmt*, and *Gsta2/m6/z1/Mgst3*, respectively).
- Transport reactions, mediated by DTPs that have a role in the facilitated carrying of drugs across cellular membranes (Katz et al., 2008; Penner et al., 2012). The eQTL targets in this class include two types of DTP families: an ATP-binding cassette (*Abcc6*) and a solute-linked carrier (*Slco1a1*).
- Transcription regulation. The identified eQTL targets include CAR, a nuclear receptor that regulates the transcription of drug disposition enzymes.

**Table 1:** **Summary of proximal eQTLs underlying the biotransformation of drugs in livers of the CC mouse population**.

**Reaction**	**Enzyme class**	**Enzyme**	**eQTL**
Functionalization: oxidation	Cytochromes P450 (CYPs)	*Cyp2a22*	chr7:28
		Cyp2c40^*^	chr19:40
		*Cyp2c44*	chr19:40
		*Cyp2d11*	chr15:84
		*Cyp2d12*	chr15:86
		*Cyp3a13*	chr5:138
		*Cyp3a16*	chr5:147
		Cyp3a25^**^	chr5:147
	Alcohol dehydrogenase	Adhfe1^*^	chr1:12
	Aldehyde dehydrogenase	*Aldh8a1*	chr10:22
		*Aldh16a1*	chr7:50
	Thiol-disulfide oxidoreductase	Glrx2^**^	chr1:146
	Flavin-containing monooxygenase (FMO)	*Fmo1*	chr1:160
Functionalization: reduction	Aldo-keto reductase	*Akr1c13*	chr13:3
		*Akr1c19*	chr13:5
Functionalization: hydrolysis	Carboxylesterase (CES)	*Ces1g*	chr8:99
		*Ces2h*	chr8:112
		Ces3a^*^	chr8:108
	Sialic acid acetylesterase	*Siae*	chr9:40
	Sulfatase (esterase)	*Sulf2*	chr2:167
	Epoxide hydrolase	*Ephx2*	chr14:62
	Dihydropyrimidinase	Dpys^*^	chr15:39
	Glucuronidase	Gusb^*^	chr5:130
	Glyoxalase	Glo1^**^	chr17:32
Conjugation	UDP-glucuronosyltransferase (UGT)	Ugt1a6a^*^	chr1:92
		Ugt1a6b^**^	chr1:90^*^,92
		*Ugt1a10*	chr1:92
		*Ugt3a1*	chr15:11
		*Ugt3a2*	chr15:9
	Methyltransferase	Tpmt^**^	chr13:51
	N-acetyl transferase	*Nat8*	chr6:86
	Amino acid (AA) transferase	Ccbl1^*^	chr2:30
		Ccbl2^*^	chr3:143
	Glutathione S-transferase (GST)	*Mgst3*	chr1:170
		Gsta2^*^	chr9:82
		*Gstm6*	chr3:105
		*Gstz1*	chr12:90
Transport	ATP-binding cassette	*Abcc6*	chr7:51
	Solute-linked carrier	*Slco1a1*	chr6:142
Transcription factors	Nuclear receptor	Nr1i3 (CAR)^*^	chr1:174

*Shown for each drug disposition reaction (column 1) are various enzyme classes (column 2), the identified eQTL-associated genes in each class (column 3), and the top-ranking proximal eQTL of each gene (genomic positions; column 4. The second number indicates the distance in Mb from the start of the chromosome). Expression of each enzyme is associated with an isoform-ratio eQTL (asterisk), total-expression eQTL (no asterisk), or both (two asterisks). Overall, we found 30 total-expression eQTLs and 15 isoform-ratio eQTLs that underlie the expression of 40 different drug disposition enzymes*.

### Mapping the connectivity between eQTLs and drug metabolism

**Figure 1:**
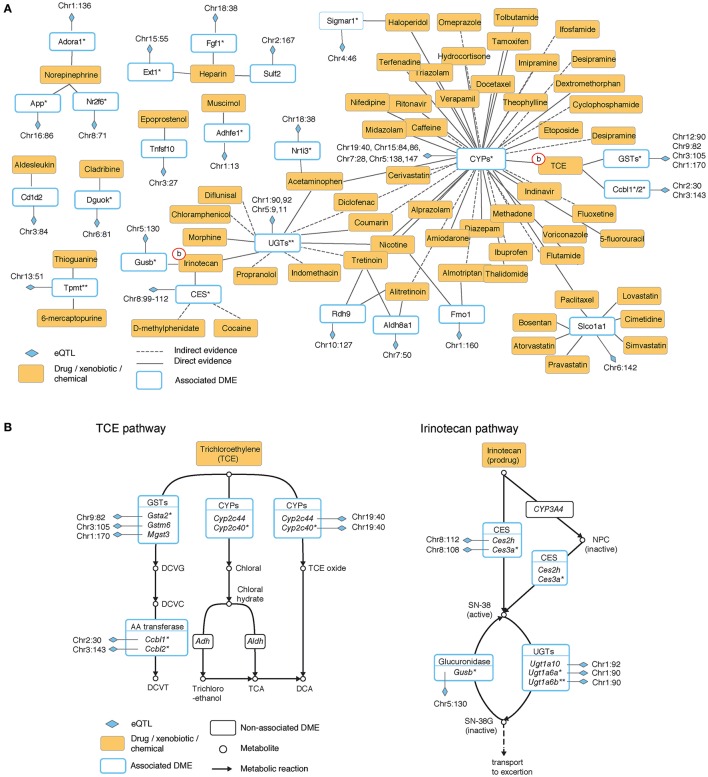
**The hepatic eQTL-drug connectivity map reveals the organization of proximal regulatory variants acting on drug disposition processes. (A)** Hepatic eQTL-drug connectivity map. A network view of exogenous chemicals and drugs (orange nodes) and drug disposition enzymes (white nodes with blue borders) with significant association to proximal eQTLs (blue diamonds). Edges correspond to a known role of an enzyme in the metabolic biotransformation of a given chemical. Solid or dashed lines indicate direct or indirect evidence, respectively. **(B)** Zoom-in on the underlying metabolic reactions of two representative exogenous chemicals: trichloroethylene (TCE, left) and irinotecan (right). Chemicals and drugs are shown as orange rectangles; enzymes are shown as white rectangles; eQTLs are marked by blue diamonds, and eQTL-associated enzymes are drawn with a blue border. Plot **(A)** summarizes the eQTL-drug connectivity in these pathways. Shown are isoform-ratio eQTL (asterisk), total-expression eQTL (no asterisk), or both (two asterisks). CYPs, cytochromes P450; CES, carboxylesterase; UGTs, UDP-glucuronosyl transferases; GSTs, glutathione S-transferases.

To obtain a global perspective on the participation of eQTLs in drug metabolism we used expert-curated drug-specific sets of enzymes, where each set is a collection of enzymes that play a role in the biotransformation of one particular drug (see Materials and Methods). By analyzing these sets we identified 63 drugs whose biotransformation is perturbed by at least one proximal eQTL (Table S5) and of which 49 are affected by two or more eQTLs that reside in a distinct genomic location (at least 10 Mb apart; Table S6). We organized this information as a network, referred to as the “eQTL-drug connectivity map” (Figure 1A). The map is composed of three types of nodes: drugs, eQTLs and enzymes. Each drug is connected to its metabolizing enzyme nodes and each enzyme is connected to its underlying eQTL nodes.

Characterization of drugs in terms of their perturbing eQTLs provides potential joint effects that may underlie the response to administration of a drug. For example, trichloroethylene (TCE) is a small molecule (C_2_HCl_3_) with carcinogenic potential. In the connectivity map, TCE is connected to three eQTL targets (cytochrome P450 [CYPs], glutathione S-transferases [GSTs] and amino acid [AA]-transferases). The connectivity map is based on the known biochemical pathway involving the same combination of eQTL targets (Figure 1B, left). The biochemical pathway suggests a potential process by which joint genetic effects may influence the response to TCE.

The irinotecan pathway provides another example of potential genetic interactions within the eQTL-drug connectivity map (Figure 1B, right). Irinotecan is a drug used for the treatment of cancer (Nagar and Blanchard, 2006), and there are known individual differences in susceptibility to its effect (Nagar and Blanchard, 2006; Guo et al., 2007; Marsh and Hoskins, 2010). The pathway consists of activation of irinotecan to SN-38 (by members of the carboxylesterases [CES] family) and deactivation of SN-38 into SN-38G (by the UDP-glucuronosyltransferase [UGTs] and the glucuronidase families). Overall, whereas the eQTL-drug connectivity map (Figure 1A) summarizes the identity of the relevant metabolizing enzymes, mechanistic visualization (Figure 1B) suggests the existence of genetic interactions between different eQTLs along the cascade of metabolic reactions.

In addition, the connectivity map indicates that some of the associated enzymes (8 of 23; 34%) participate in the biotransformation of two or more drugs, highlighting potential regulatory variation that may lead to drug-drug interactions. One example is the solute-linked carrier *Slco1a1*, whose proximal regulatory variation probably has an effect on at least seven drugs, including lovastatin, bosentan and cimetidine. Another example is the family of UDP-glucuronosyl transferases (UGTs), which is connected to 11 different drugs. This suggests specific proximal regulatory variation that has an influence on a large repertoire of drugs.

We note that it is possible to identify groups of CC lines on the basis of their co-variation in the expression of drug-specific metabolizing enzymes. For example, as in the case of the irinotecan pathway (Figure S7), there is a clear grouping of the CC lines based on the co-variation of their expression across the relevant enzymes (e.g., a distinct expression of lines IL-670 and IL-785 compared to lines IL-611 and IL-3438 across the expression of *Ces2h/3b, Ugt1a10/6a/6b*, and *Gusb*). Based on this grouping it is possible to select a non-redundant subset of lines that covers a large variety of responses (e.g., by choosing a single line from each group). Because distinct drugs initiate different biotransformation pathways perturbed by distinct combinations of eQTLs, this strategy may provide a tailored drug-specific selection of CC lines, opening the way to future expression-based selection of representative CC lines that can be used to test adverse effects in pharmacogenetic studies.

### Genetic variation in alternative splicing of hepatic drug disposition enzymes

Our data showed that a substantial fraction of the identified associations are a result of splicing variability. Among 608 associated traits, we found 243 isoform-ratio traits (~40%; Tables S3, S4). Furthermore, we observed a similar percentage of isoform-ratio traits within the associated drug deposition genes (33%, 15 from 45 traits; Table 1). In the TCE pathway, for instance, out of seven associated genes we identified four (57%) whose association was due to variation in splicing events spanning four different metabolic transformations (ending with TCE oxide, chloral, DCVG and DCVT; Figure 1B, left). Similarly, among six associated genes in the irinotecan pathway, four are controlled at the level of alternative splicing (*Ces3a, Gusb, Ugt1a6a/b*, 66%; Figure 1B, right). These results are comparable with the reported isoform-ratio percentages of 38% (496 out of 1290) and 44% (529 out of 1191) in human Caucasian and Yoruba cohorts (60 and 69 individuals, respectively, Gonzàlez-Porta et al., 2012), and are in agreement with studies indicating that isoform-ratio eQTLs are prevalent but less abundant than total-expression eQTLs (e.g., Battle et al., 2014).

We then turned to characterizing representative examples of isoform-level associations. For each eQTL-associated gene we first selected founder strains that differ substantially in their effects, and then chose CC lines that carry the haplotype of the selected founders in the associated locus (eQTL) of the gene. We note that CC lines for a gene are selected because they carry the genetic information of founders, which leads to a major variation in the fraction of expression of at least one isoform; the selected CC lines should therefore show a distinct distribution of the spliced junctions. Here we focus on two genes, *Cyp3a25* and *Gsta2*. For each of these genes we first exemplify the abovementioned CC selection procedure and then show the splice junctions in each of the selected strains.

**Figure 2:**
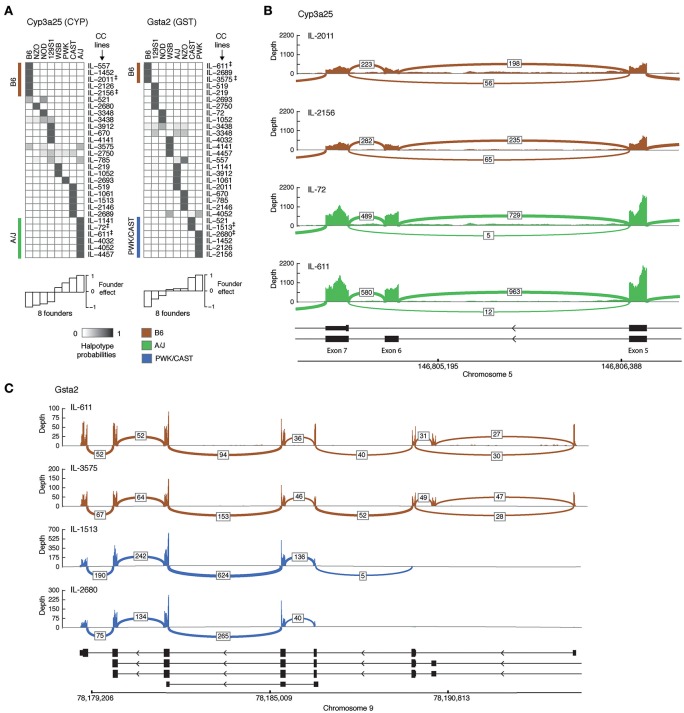
**Genetic variation in alternative splicing of drug disposition enzymes. (A)** Haplotype probabilities of the eight founder lines (columns) in 29 CC lines (rows), calculated in the isoform-ratio eQTLs of two drug disposition genes, *Cyp3a25* (left) and *Gsta2* (right). The gray scale indicates haplotype probabilities, ranging between zero (white) and 1 (dark gray). The calculated effect of each founder is shown in white bars (bottom). Groups of CC lines with the largest (positive and negative) founder effect of their haplotype are marked in brown and green (B6- and A/J-carrying lines; *Cyp3a25*) or brown and blue (B6- and CAST/PWK-carrying lines; *Gsta2*). Double daggers indicate two representative CC lines in each of these groups, which were used for displaying the raw sequencing reads in plots **(B,C)**. **(B,C)** Raw reads of selected strains for the genes Cyp3a25 and Gsta2. The read‘s coverage over exon is displayed as bar graph, and the number of reads across splice junctions (junction depth) are displayed by arcs. Arcs with junction depth < 5 were omitted. The known isoforms are indicated in black (bottom). **(B)** The *Cyp3a25* locus, focusing on exons 5, 6, and 7 in CC individuals that carry the B6 haplotype (brown) and the A/J haplotype (green) in the associated eQTL. **(C)** Entire *Gsta2* locus (excluding exon 1 of the longest isoform) in CC individuals that carry the B6 haplotype (brown) and the PWK or CAST haplotype (blue) in the associated locus. B6, C57BL/6J; 129S, 129S1/SvIm; NOD, NOD/ShiLtJ; WSB, WSB/EiJ; NZO, NZO/HILtJ; CAST, CAST/EiJ; PWK, PWK/PhJ.

1. Cyp3a25 is a functionalization enzyme that oxidizes a variety of compounds including xenobiotics and steroids. We found that the founder B6 and A/J strains differ substantially in their effects on at least one isoform of this gene (Figure 2A, bottom left). We therefore distinguish between CC lines that carry the B6 haplotype (IL-557, IL-1452, IL-2011, IL-2126, IL-4156 strains) and those carrying the A/J haplotype (IL-1141, IL-72, IL-611, IL-4032, IL-4052, IL-4457 strains) in the genomic region of Cyp3a25 (Figure 2A, left). Specifically, we focus on two representative B6-carrying lines (IL-2011 and IL-2156) and two A/J-carrying lines (IL-72 and IL-611). Figure 2B displays the read coverage of the exons and the numbers of junction reads of the selected strains. We found multiple reads aligned to the junction-spanning exons 5 and 7 in B6-carrying strains (56 and 65 reads in lines IL-2011 and IL-2156, respectively). Notably, the numbers of these exon-skipping reads was substantially lower in the A/J-carrying individuals (5 and 12 reads in lines IL-72 and IL-611, respectively). The widespread exon-skipping events observed in B6-carrying strains raise the possibility that these events reflect a higher level of expression in B6-carrying than in A/J-carrying strains. However, we observed the opposite effect, with a higher coverage level and a higher number of non-skipping splicing reads in the A/J-carrying individuals. For instance, 729 and 963 junction reads between exons 6 and 7 were mapped in the two A/J-carrying strains compared to 198 and 235 junction reads in B6-carrying ones. This confirms that the larger numbers of exon 6-skipping events in B6-carrying strains are not merely a result of the overall level of expression.
2. Gsta2 is a conjugation enzyme that adds a reduced glutathione to hydrophobic electrophiles. Relying on the observation that the founder effect of the B6 line differs substantially from the PWK and CAST lines (Figure 2A, right), we focused on two B6-carrying CC lines (IL-611 and IL-3575), one CAST-carrying line (IL-1513) and one PWK-carrying line (IL-2680; Figure 2C). We observed alternative 5′ start sites in which only the B6-carrying individuals have alternative upstream start sites. This could not be ascribed merely to the higher overall expression of *Gsta2* in the B6-carrying individuals, since the coverage of reads (across all exons) is lower in B6-carrying than in CAST/PWK-carrying individuals. The difference between the haplotypes can be attributed to an alternative location of the 5′ start site, or alternatively, to a haplotype-specific 5′–3′ RNA degradation. Previously annotated isoforms do not fully explain the observed reads in the PWK and CAST CC lines.

### Substantial transcriptional diversity in the crosstalk between drug metabolism and lipids metabolism

**Figure 3:**
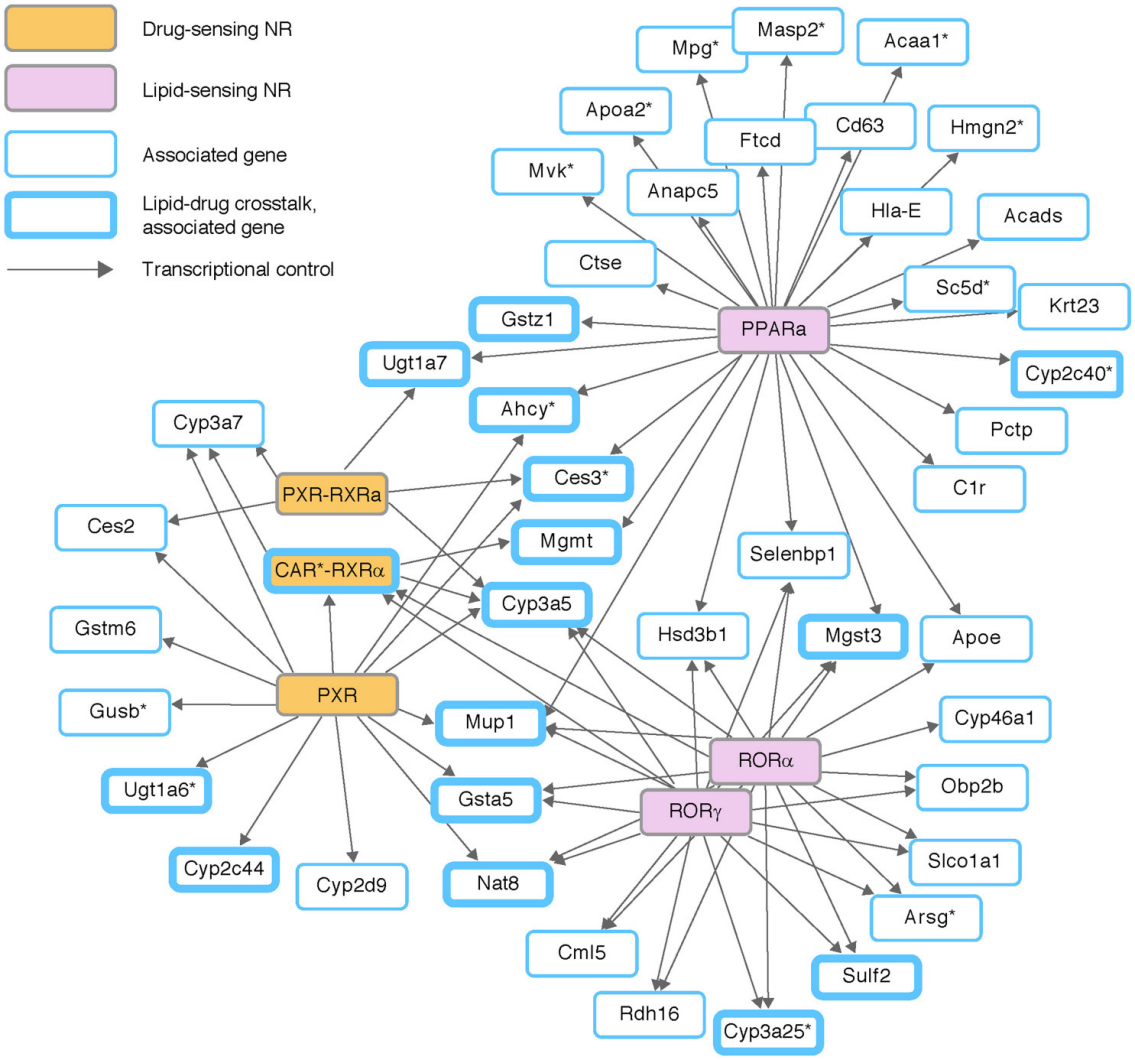
**Genetic variation in the crosstalk between drug and lipid metabolism**. Three chemical and drug-sensing nuclear receptors (NRs; orange nodes) and three lipid-sensing NRs (pink nodes), shown together with their transcriptional regulation (edges) on eQTL-associated target genes (blue-border nodes). Targets in the crosstalk between drug and lipids metabolism (either based on prior knowledge or based on the transcriptional control in this network) are drawn with thickened border. Out of 46 NR-dependent eQTL targets, 16 targets are involved in the lipid-drug crosstalk.

We next analyzed regulatory programs of ligand-activated transcription factors called nuclear receptors (NRs). NRs were selected not only because they play a major role in transcription regulation, but also since many DMEs and DTPs are induced by their own substrates through the activity of NRs. Chemical signals (ligands) of NRs consist of exogenous drugs and xenobiotics, as well as endogenous small molecules such as steroid hormones and cholesterol (e.g., Evans and Mangelsdorf, 2014). Using the extensive mapping of regulatory programs within the curated IPA, we identified seven different NRs (or receptor complexes) whose targets are enriched among the 608 associated traits (Figure 3). Specifically, we found enrichment of targets in xenobiotics- and drug-activated NRs, including the xenobiotics sensor PXR—with or without its heterodimerization partner RXRα (*P* < 0.0024, 0.0158, respectively; hyper-geometric test)—and the xenobiotics sensor CAR as an heterodimer with RXRα (*P* < 0.0165). We also found that the hepatic eQTL targets were enriched with known targets of RORα, RORγ, and PPARα, three NRs that are activated by cholesterol and fatty acid ligands (*P* < 0.0012, 0.0012, 0.0081, respectively).

The map of NR-dependent eQTL targets shows a clear overlap between targets of xenobiotics-sensitive regulators and targets of lipid-sensitive regulators (Figure 3). Specifically, six of 16 genes that are regulated by xenobiotics sensors (PXR and CAR) are also regulated by lipid sensors (RORα, RORγ, and PPARα). A close examination of the map showed that the overlap between lipid metabolism and drug metabolism is even more prominent: the known DME genes *Sulf2, Cyp3a25, Cyp2c40, Gstz1*, and *Mgst3* are targeted by lipid-sensing nuclear receptors; similarly, two genes that are controlled by xenobiotics-activated receptor are well-established lipid metabolizing enzymes (*Ugt1a6* and *Cyp2c44*).

These results are consistent with known biochemical reactions that may act on unrelated compounds. For example, Cyp3a16 has a role in degradation of steroids, in addition to its role in oxidation of a variety of drugs, including tamoxifen, paclitaxel, haloperidol and tretinoin; similarly, Ugt1a6b participates in hepatic degradation of C_18_-steroids and also in conjugation of levothyroxine, nicotine and irinotecan (Figure 1A). Furthermore, our analysis is consistent with previous reports, both in human and mouse, where disordered lipid metabolism has an effect on clinical drug disposition and the other way around. For example, non-alcoholic fatty liver disease (NAFLD) patients exhibit altered metabolism of drugs (Buechler and Weiss, 2011), and DTPs are down-regulated in non-alcoholic steatohepatitis (NASH) subjects (Lake et al., 2011). Furthermore, several genetic modifiers of NAFLD and NASH have a documented effect on the efficacy of drugs (reviewed in Naik et al., 2013). Our results in the CC panel demonstrate the prevalence of genetic variation acting on the lipid-drug crosstalk. CC mice can therefore be used to investigate the relationships between these factors and their roles in the interaction between lipid metabolism and metabolism of drugs and xenobiotics.

## Discussion

A central challenge in pharmaceutical research has been to investigate genetic variation in response to drugs, chemicals and xenobiotics. The panel of CC lines is a promising model for pharmacogenomic studies because of the large amount of genetic variation and the ability to investigate the molecular response to drugs within specialized internal tissues. However, the effect of genetic variation on drug disposition enzymes remain to be elucidated. Here we defined the diversity and complexity of genetic variation on the expression of drug disposition genes across the CC lines. Our results indicate a previously unknown overrepresentation of hepatic drug disposition genes that are affected by proximal regulatory variation (*P* < 0.03; Table 1). Further inspection showed the complexity of inter-relationships between regulatory variants, highlighting various potential interactions between drugs due to shared eQTLs (Figures 1–3). This analysis, therefore, provides an informative view to guide future pharmacogenetics and mechanistic studies across the CC strains. For example, we found a significant effect of lipid metabolism on pharmacokinetic parameters (Figure 3). This suggests that the CC mice are a suitable model for studying the lipid-drug crosstalk in human metabolic disorders.

Measuring toxicity and adverse effects across a large population of genetically distinct individuals is costly. Our analysis offers a potential strategy for selecting a subset of CC lines (designed for a specific drug) that can be used in pharmacogenomic studies. In particular, a given drug corresponds to a group of eQTL targets that play a role in deposition of the drug (e.g., six DMEs in the irinotecan pathway; Figure 1B). The transcription profiles of CC lines across the genes in this group may point to a small subset of non-redundant CC lines (e.g., for the irinotecan pathway, a single CC line from each of the eight groups in Figure S7). In subsequent pharmacogenetic studies of this drug, only this subset of CC lines is analyzed. This strategy is in contrast to the standard approach of choosing the lines according to genotyping. The two selection methods complement each other, but the expression-based approach may reveal, in addition, the functional effects of candidate genomic loci.

Although it is not yet known whether the selection of CC mice based on proximal eQTLs reflects drug toxicity and adverse effects *in vivo*, it provides a plausible strategy of a rationalized selection of strains that (i) relies on variation in transcript isoforms in addition to the overall expression of genes; (ii) is tailored to each specific drug; and (iii) allows selection of additional CC mice not included in the reference database (e.g., using the inferred trait levels based on the optimized parameters in Equation 1). Future studies should determine whether an expression-based CC selection strategy is predictive of drug toxicity and adverse outcome. A more complete approach will require integration with additional genomic data, including proteomics, *trans*-acting polymorphic loci, and epigenetic data.

## Author contributions

All authors listed, have made substantial, direct and intellectual contribution to the work, and approved it for publication.

## Funding

This work was supported by the European Commission [FP7/2007-2013, under grant agreement no. 262055 (ESGI)], by the Israeli Centers of Research Excellence (I-CORE): Center No. 41/11, by the Broad-ISF grant 1168/14, and by the Wellcome Trust grants 090532/Z/09/Z, 085906/Z/08/Z, 083573/Z/07/Z, and 075491/Z/04. Research in the IG lab was supported by the European Research Council (637885), and by the Israeli Science Foundation (ISF) grant 1643/13. YS was partially supported by the Edmond J. Safra Center for Bioinformatics at Tel Aviv University IG is a Faculty Fellow of the Edmond J. Safra Center for Bioinformatics at Tel Aviv University and an Alon Fellow. RS was supported in part by the Raymond and Beverly Sackler Chair in Bioinformatics.

### Conflict of interest statement

The authors declare that the research was conducted in the absence of any commercial or financial relationships that could be construed as a potential conflict of interest.
