# Supplementary material for: Dissecting the Effect of Genetic Variation on the Hepatic Expression of Drug Disposition Genes across the Collaborative Cross Mouse Strains
Source: Front Genet. 2016 Oct 5;7:172. doi: 10.3389/fgene.2016.00172 (PMC5050206; doi:10.3389/fgene.2016.00172)
Supplement: Supplementary file 1 [file DataSheet1.PDF]

*Supplementary Material:*

# Dissecting the effect of genetic variation on the hepatic expression of drug disposition genes across the collaborative cross mouse strains

**Aharon Nachshon[1], Hanifa J. Abu-Toamih Atamni[2], Yael Stauerman[1], Roa'a Sheikh-Hamed[2], Alexandra Dorman[2], Richard Mott[3], Juliane C. Dohm[4,5,6], Hans Lehrach[7], Marc Sultan[7], Ron Shamir[8], Sascha Sauer[7,9,10], Heinz Himmelbauer[4,5,6], Fuad A. Iraqi[2*†], Irit Gat-Viks[1*†]**

[1]Department of Cell Research and Immunology, Faculty of Life Sciences, Tel-Aviv University, 69978 Israel. [2]Department of Clinical Microbiology and Immunology, Sackler Faculty of Medicine, 69978 Tel-Aviv University, Israel. [3]Genetics Institute, University College of London, London, UK. [4]Center for Genomic Regulation, C/ Dr. Aiguader, 88, 08003 Barcelona, Spain. [5]Universitat Pompeu Fabra (UPF), C/ Dr. Aiguader, 88, 08003 Barcelona, Spain. [6]Department of Biotechnology, University of Natural Resources and Life Sciences Vienna (BOKU), Muthgasse 18, 1190 Vienna, Austria. [7]Department of Vertebrate Genomics, Max Planck Institute for Molecular Genetics, Ihnestr. 63–73, 14195 Berlin, Germany. [8]The Blavatnik School of Computer Science, Tel Aviv University, 69978 Tel Aviv, Israel. [9]CU Systems Medicine, University of Würzburg, 97080 Würzburg, Germany. [10]Current address: BIMSB and BIH Genomics Platforms, Laboratory of Functional Genomics, Nutrigenomics and Systems Biology, Max-Delbrück-Center for Molecular Medicine, Robert-Rössle-Straße 10, 13125 Berlin, Germany.

[*] To whom correspondence should be addressed: iritgv@post.tau.ac.il and fuadi@post.tau.ac.il

[†] These authors contributed equally to this work

**SUPPLEMENTARY TABLES AND FIGURES**

**1. Figures**

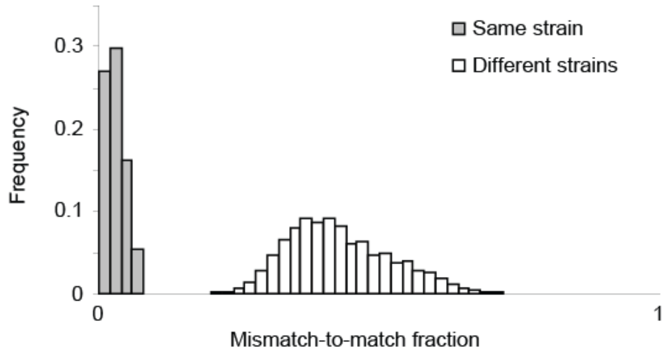


**Figure S1: Validation of genotyping data**. The quality of genotyping was assessed using comparison between a standard array-based genotyping versus an RNA-Seq-based genotyping. Differences between the inferred genotypes (genotyping array against RNA-Seq-based genotyping) were assessed using the 'mismatch-to-match fraction' metric - defined as the fraction of mismatches to matches across all SNPs located in transcribed regions. The plot presents the distribution of mismatch-to-match fraction when using the *same* strain (gray), as well as the distribution of mismatch-to-match fraction when using one strain for the RNA-Seq-based genotyping and *another* strain for the array-based genotyping (white). The results indicate that the genotyping arrays are highly accurate (that is, low values of mismatch-to-match fraction when using the same strain).

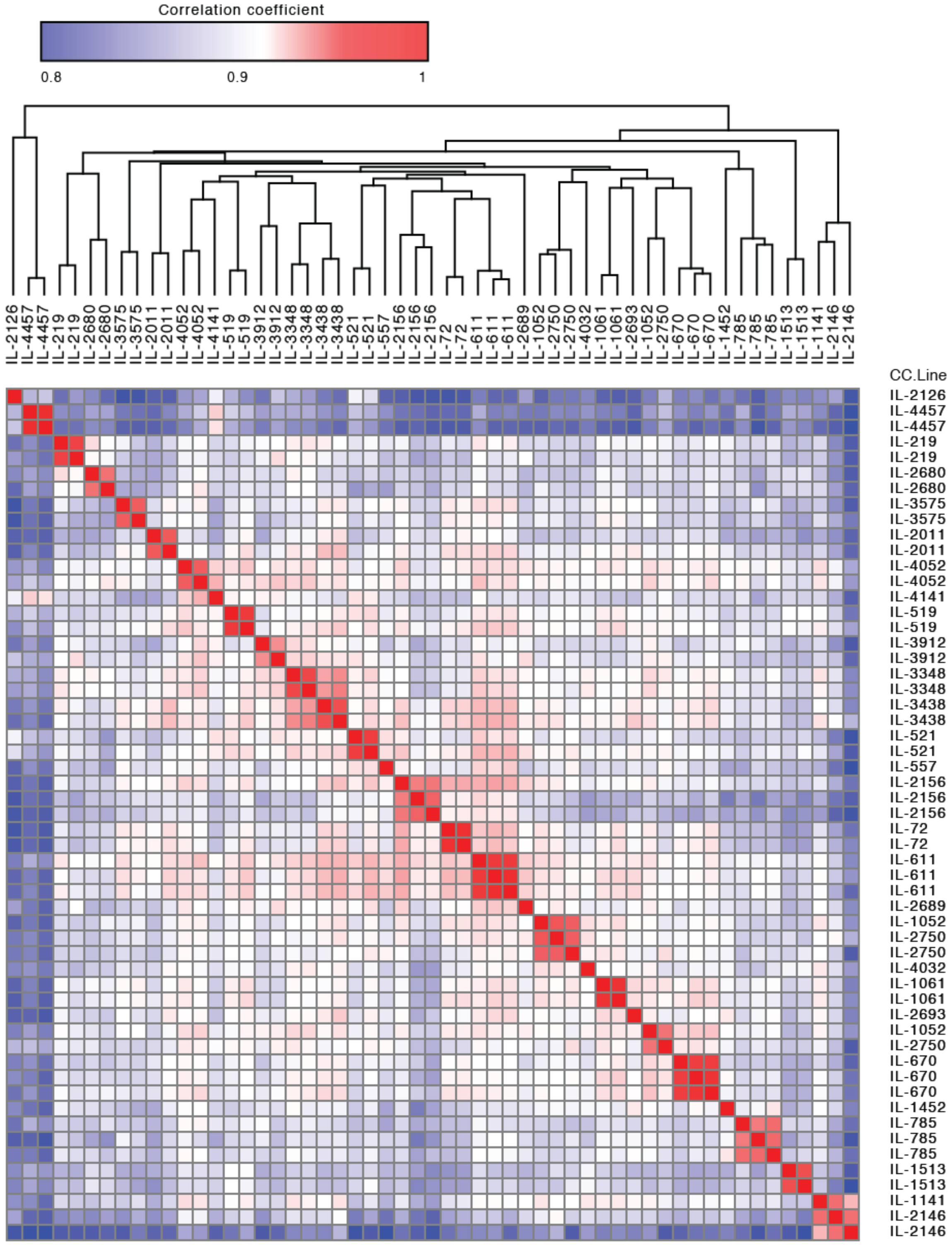


**Figure S2**. **Global analysis of total-expression traits.** Clustered correlation matrix of all CC individuals. The Spearman correlation coefficients (red blue color bar) was calculated using the total-expression values.

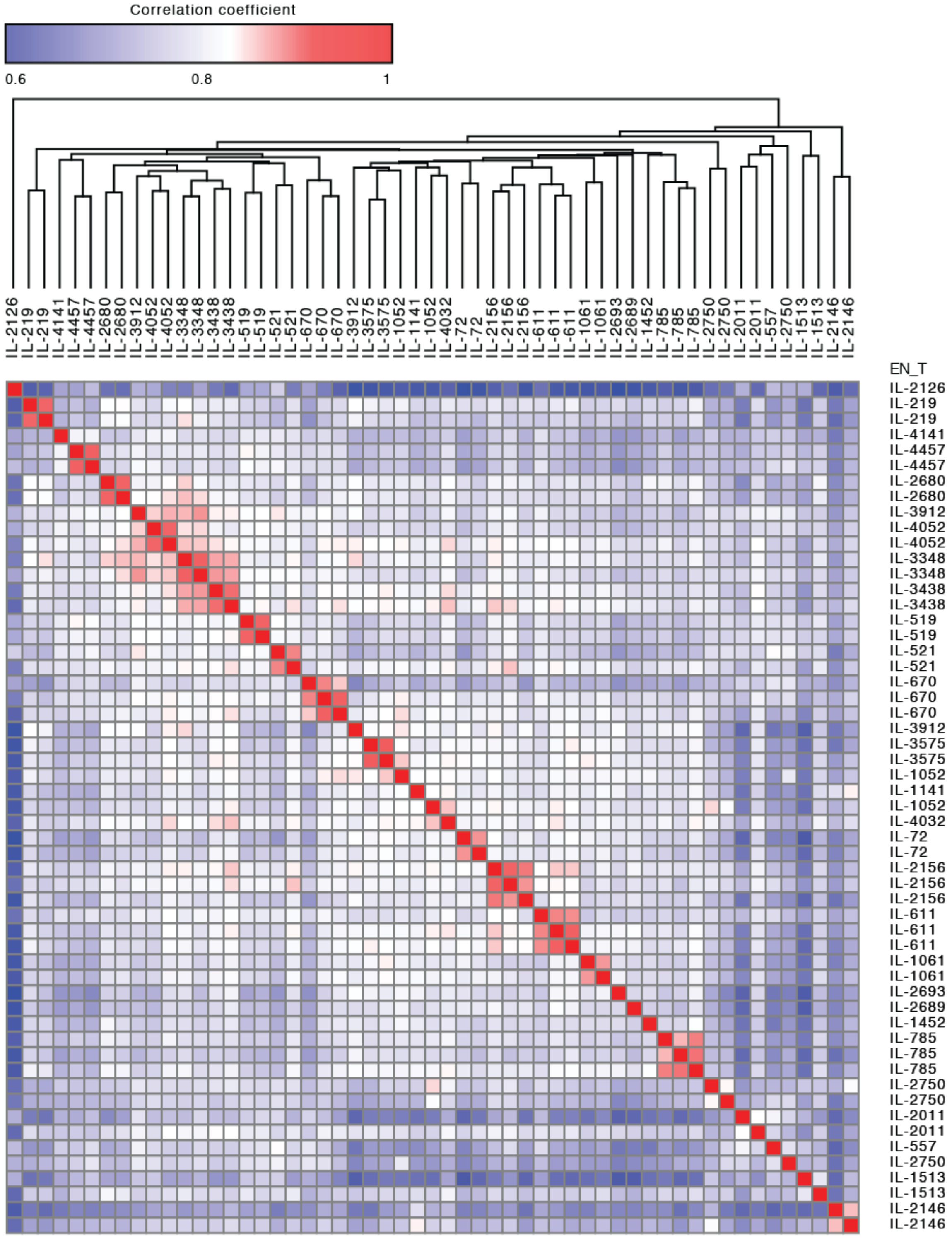


**Figure S3**. **Global analysis of isoform-ratio traits**. Clustered correlation matrix of all CC individuals. The Spearman correlation coefficients (red blue color bar) was calculated using the isoform-ratio values.

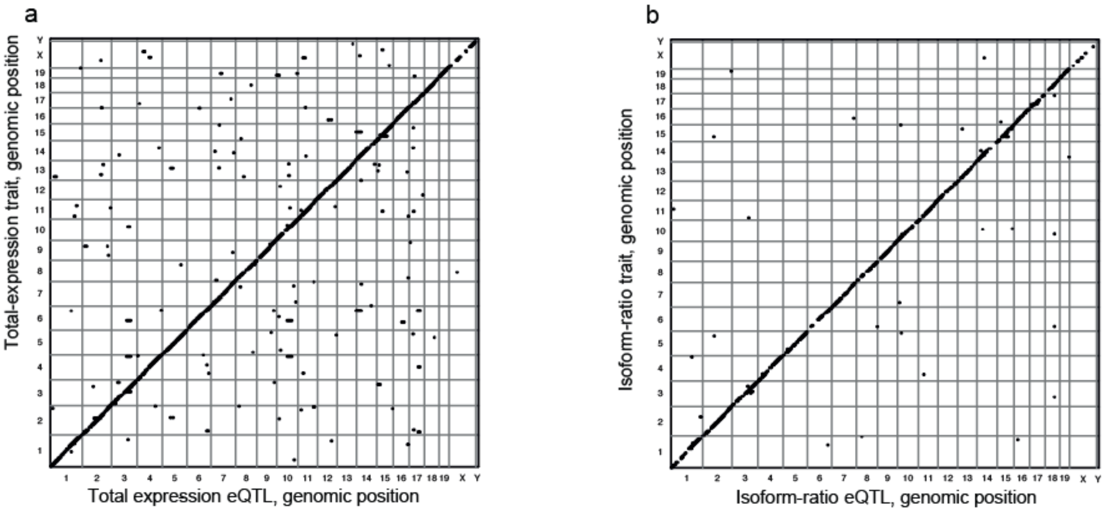


**Figure S4: Genome–genome plots of total-expression eQTLs and isoform-ratio eQTLs**. Shown are eQTL maps of a genome-wide eQTL analysis that was applied on the entire genome (without limitation to 5 Mbp), using an FDR cutoff of 0.1, for both total-expression traits (**a**) and isoform-ratio traits (**b**). In these eQTL maps, each point indicates the genomic position of the best-scoring variant (*x*-axis) and the position of its associated target (*y*-axis). We note that vertical bands in this map are typically induced by technical confounding factors (rather than by a regulatory hotspot, as demonstrated by Kang et al., 2008). Plots (**a**) and (**b**) demonstrate the absence of such vertical bands, providing an evidence for the validity of our results.

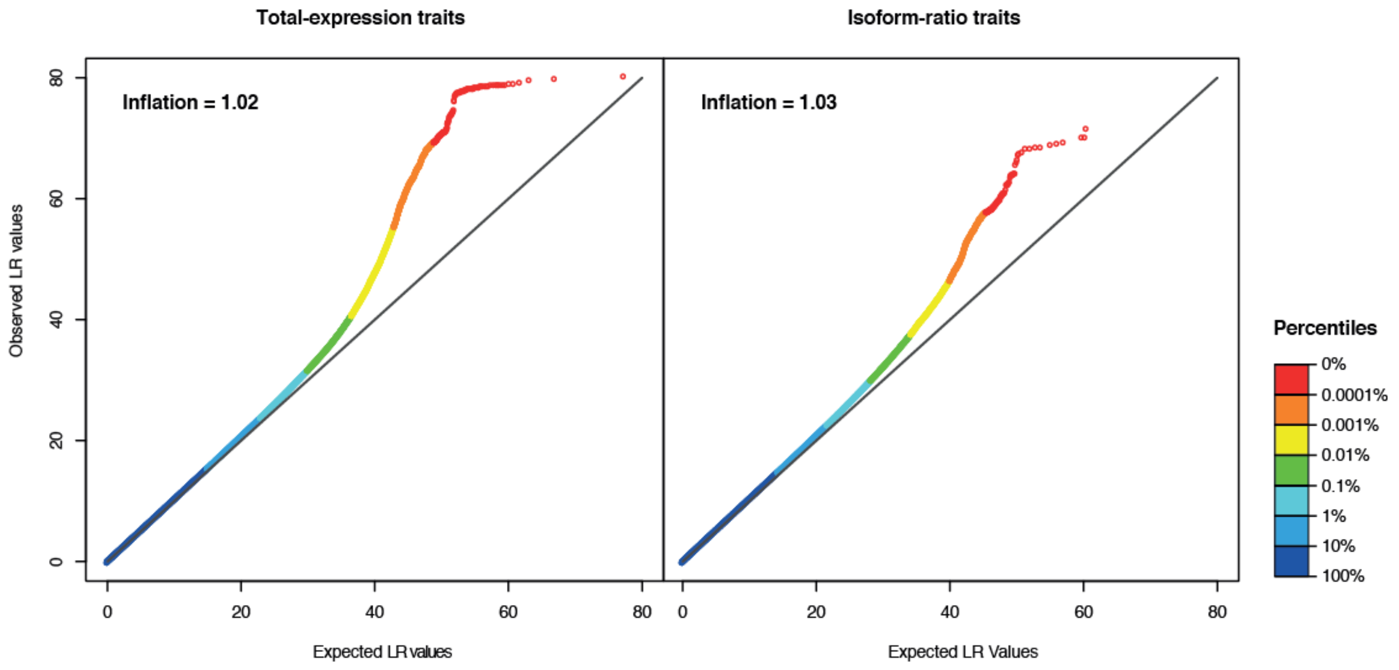


**Figure S5**: **Quantile-quantile (QQ) plots of total-expression and isoform-ratio traits**. The QQ plots present the null distribution of LR values (*x*-axis) compared to the distribution of observed LR values (*y*-axis). Results are shown across all total-expression traits or isoform-ratio traits (left and right, respectively) and across all SNPs. In both cases the inflation factor (the ratio of the median of the observed distribution to the median of the expected distribution) is smaller than 1.03, indicating that the identified eQTLs in this study are not due to confounding effects. Traits are color coded according to the percentile of their observed LR values.

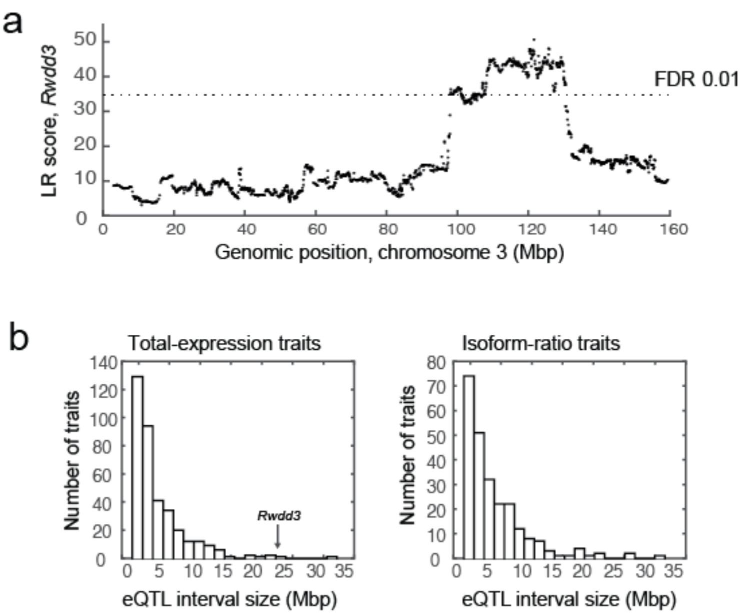


**Figure S6: Imprecision of association**. For each trait, its 'eQTL interval size' is the length of the proximal genome region that attained association scores exceeding an FDR of 0.01. (**a**) A representative example of a significant eQTL interval. Shown are the association scores (LR values; *y*-axis) across genomic positions in chromosome 3 (*x*-axis) for the total-expression of *Rwdd3*. The plot clearly demonstrates the large eQTL interval of *Rwdd3* (23.6 Mbp, between chr3:106,932,696 and chr3:130,535,749.5). (**b**) The distribution of eQTL interval sizes across the 365 total-expression traits (left) and the 243 isoform-ratio traits (right). The eQTL interval size of *Rwdd3* is marked with an arrow.

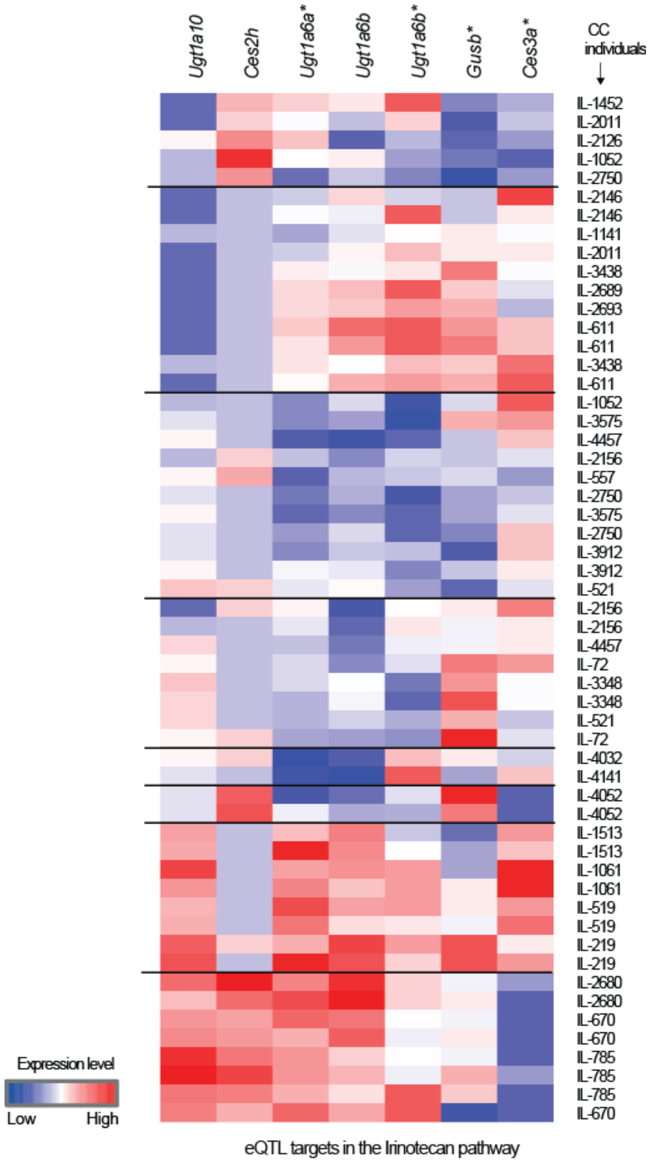


**Figure S7. Expression profiles of CC mice across DMEs with proximal eQTLs in the Irinotecan pathway.** The matrix presents the expression level of eQTL-associated traits within the Irinotecan pathway (columns) across different CC individuals (rows). High and low expression levels are in red and blue, respectively. Shown are three total-expression traits (*Ces2h*, *Ugt1a10* and *Ugt1a6b*) and four isoform-ratio traits (*Ces3a*, *Ugt1a6a*, *Gusb* and *Ugt1a6b*; indicated with asterisks). Each individual is annotated with the identifier of its CC strain. The clustering of the rows into eight groups is indicated as vertical lines.

## 2. Tables

**Table S1. Liver samples.** For each individual mouse (column 1), the table presents the CC line (column 2), age (weeks, column 3) and weight (gr; column 4).

| CC individual | CC Line | Age (weeks) | Body Weight (gr) |
|---|---|---|---|
| 1 | IL-72 | 9.4 | 23.0 |
| 2 | IL-72 | 9.4 | 24.0 |
| 3 | IL-219 | 9.1 | 21.7 |
| 4 | IL-219 | 9.1 | 18.5 |
| 5 | IL-519 | 8.0 | 19.9 |
| 6 | IL-519 | 8.0 | 21.5 |
| 7 | IL-521 | 9.9 | 23.2 |
| 8 | IL-521 | 9.9 | 24.5 |
| 9 | IL-557 | 8.4 | 23.0 |
| 10 | IL-611 | 10.1 | 28.4 |
| 11 | IL-611 | 10.1 | 26.9 |
| 12 | IL-611 | 10.1 | 29.5 |
| 13 | IL-670 | 9.1 | 20.2 |
| 14 | IL-670 | 9.1 | 19.6 |
| 15 | IL-670 | 9.1 | 21.8 |
| 16 | IL-785 | 8.1 | 15.8 |
| 17 | IL-785 | 8.3 | 15.4 |
| 18 | IL-785 | 9.0 | 12.8 |
| 19 | IL-1052 | 9.3 | 23.6 |
| 20 | IL-1052 | 9.3 | 22.3 |
| 21 | IL-1061 | 8.3 | 17.7 |
| 22 | IL-1061 | 8.3 | 19.6 |
| 23 | IL-1141 | 8.9 | 24.4 |
| 24 | IL-1452 | 9.9 | 22.3 |
| 25 | IL-1513 | 8.1 | 16.0 |
| 26 | IL-1513 | 8.1 | 17.8 |
| 27 | IL-2011 | 9.4 | 21.3 |
| 28 | IL-2011 | 9.4 | 21.6 |
| 29 | IL-2126 | 10.9 | 17.8 |
| 30 | IL-2146 | 9.3 | 18.4 |
| 31 | IL-2146 | 9.3 | 21.6 |
| 32 | IL-2156 | 10.0 | 38.0 |
| 33 | IL-2156 | 10.0 | 27.7 |

| | | | |
|---|---|---|---|
| 34 | IL-2156 | 10.0 | 25.3 |
| 35 | IL-2680 | 11.9 | 28.3 |
| 36 | IL-2680 | 11.9 | 25.1 |
| 37 | IL-2689 | 8.1 | 22.0 |
| 38 | IL-2693 | 8.1 | 18.1 |
| 39 | IL-2750 | 8.1 | 26.9 |
| 40 | IL-2750 | 8.1 | 30.1 |
| 41 | IL-2750 | 8.1 | 26.8 |
| 42 | IL-3348 | 8.1 | 26.6 |
| 43 | IL-3348 | 8.1 | 23.5 |
| 44 | IL-3438 | 9.7 | 27.0 |
| 45 | IL-3438 | 9.7 | 25.0 |
| 46 | IL-3575 | 9.0 | 17.3 |
| 47 | IL-3575 | 9.0 | 17.9 |
| 48 | IL-3912 | 10.0 | 25.7 |
| 49 | IL-3912 | 10.0 | 26.9 |
| 50 | IL-4032 | 8.4 | 14.8 |
| 51 | IL-4052 | 10.9 | 23.4 |
| 52 | IL-4052 | 10.9 | 25.5 |
| 53 | IL-4141 | 10.4 | 23.6 |
| 54 | IL-4457 | 8.6 | 35.0 |
| 55 | IL-4457 | 8.6 | 30.7 |

**Table S2. The computational analysis pipeline**. For each step of the analysis (column 1), reported is the software name (column 2), its reference (column 3) and additional links (column 4).

| Pipeline step | Software | Reference | Link |
|---|---|---|---|
| **RNA-Seq quantification** | RSEM | Li and Dewey, 2011 | http://deweylab.github.io/RSEM/ |
| **Data transformation** | None | | |
| **Association tests** | | | |
| - Haplotype probabilities | R HAPPY package | Mott et al., 2000 | http://www.well.ox.ac.uk/happy/happyR.shtml |
| - Mixed model regression | R lme4 package | Bates et al., 2015 | https://cran.r-project.org/web/packages/lme4/index.html |
| **Analysis of splicing events** | | | |
| - Alignment | TOPHAT | Kim et al., 2013 | https://ccb.jhu.edu/software/tophat/index.shtml |
| - Visualization | IGV | Thorvaldsdóttir 2013 | http://software.broadinstitute.org/software/igv/ |
| **Enrichment analysis** | IPA | QIAGEN, CA | http://www.ingenuity.com/ |

**Table S3. Predicted total-expression eQTLs in liver**. For each total-expression trait (symbols; column 1), reported are the ensemble identifier (column 2), the genomic position of the trait (column 3), and the genomic position of its top ranking proximal total-expression eQTL (column 4). Column 5 indicates whether the gene is also significantly associated with an isoform-ratio eQTL, as detailed in **Table S4**.

| Total-expression trait | | | Total-expression eQTL interval | Isoform-ratio eQTL |
|---|---|---|---|---|
| Symbol | Ensembl identifier | Genomic position | | |
| **Bmyc** | ENSMUSG00000049086 | chr2:25562399-25563237 | chr2:14756526 - 26166145 | |
| **Cd59b** | ENSMUSG00000068686 | chr2:103910006-103931344 | chr2:91222531 - 112919736 | |
| **Cyp3a16** | ENSMUSG00000038656 | chr5:146197178-146230592 | chr5:141577494 - 148589118 | |
| **Cd1d2** | ENSMUSG00000041750 | chr3:86790508-86793455 | chr3:82595175 - 86230898 | |
| **Lcn13** | ENSMUSG00000062061 | chr2:25555563-25558846 | chr2:13547225 - 26166145 | |
| **Vnn3** | ENSMUSG00000020010 | chr10:23571268-23589649 | chr10:18060806 - 25939589 | |
| **Cyp2d11** | ENSMUSG00000068085 | chr15:82219584-82224452 | chr15:77983701 - 86626374 | |
| **Mettl21d** | ENSMUSG00000049882 | chr12:70678615-70684015 | chr12:60119746 - 72584475 | |
| **H2-Q10** | ENSMUSG00000067235 | chr17:35607034-35611508 | chr17:21774306 - 40179466 | |
| **6530401N04Rik** | ENSMUSG00000020956 | chr12:53089299-53107488 | chr12:41770182 - 55456902 | + |
| **0610012H03Rik** | ENSMUSG00000055312 | chr2:105064477-105219953 | chr2:75178001 - 107333276 | |
| **Ceacam2** | ENSMUSG00000054385 | chr7:26301061-26325023 | chr7:17179335 - 26385736 | |
| **Rpl29** | ENSMUSG00000048758 | chr9:106331785-106333899 | chr9:103301364 - 110507707 | + |
| **Mup15** | ENSMUSG00000096674 | chr4:60961073-61100775 | chr4:51473949 - 62075481 | |
| **Msrb2** | ENSMUSG00000023094 | chr2:19293067-19316603 | chr2:15280860 - 24520801 | |
| **Akr1c18** | ENSMUSG00000021214 | chr13:4131861-4149900 | chr13:3220944 - 10959686 | |
| **Manba** | ENSMUSG00000028164 | chr3:135148575-135234368 | chr3:132134304 - 141743698 | |
| **Gm4951** | ENSMUSG00000073555 | chr18:60371734-60407474 | chr18:57441750 - 62191758 | |
| **Ttc39a** | ENSMUSG00000028555 | chr4:109079228-109117350 | chr4:104091146 - 114241578 | |
| **Camk2b** | ENSMUSG00000057897 | chr11:5869647-5966365 | chr11:3277587 - 8861158 | |
| **Rpl21** | ENSMUSG00000041453 | chr5:147644466-147648608 | chr5:144455566 - 151532855 | + |
| **Poldip3** | ENSMUSG00000041815 | chr15:82956408-82979814 | chr15:81956141 - 86727218 | |
| **Lrit1** | ENSMUSG00000041044 | chr14:37868016-37878132 | chr14:27274289 - 47174572 | |
| **Nkiras1** | ENSMUSG00000021772 | chr14:19103650-19116456 | chr14:5958958 - 19150979 | |
| **5730469M10Rik** | ENSMUSG00000021792 | chr14:41807029-41827077 | chr14:41695864 - 45679115 | |
| **Fabp12** | ENSMUSG00000027530 | chr3:10244209-10301183 | chr3:9819605 - 12083493 | |
| **Ugt3a2** | ENSMUSG00000049152 | chr15:9265305-9300710 | chr15:6562190 - 10587824 | |
| **Ugt1a10** | ENSMUSG00000090165 | chr1:89951963-90115579 | chr1:77680615 - 95825573 | |
| **Amd2** | ENSMUSG00000063953 | chr10:35428497-35431697 | chr10:27594228 - 38447096 | |
| **5033411D12Rik** | ENSMUSG00000055137 | chr13:16949689-17786566 | chr13:15649552 - 20208147 | |
| **Tmem19** | ENSMUSG00000069520 | chr10:114777795-114799318 | chr10:111882514 - 122588223 | |
| **RP23-381B19.7** | ENSMUSG00000093483 | chr11:83105201-83108134 | chr11:81945493 - 90361138 | + |
| **Pttg1** | ENSMUSG00000020415 | chr11:43233752-43239753 | chr11:42218922 - 43922341 | |
| **Rwdd3** | ENSMUSG00000028133 | chr3:120858318-120874613 | chr3:106932696 - 130535749 | |
| **Tmem9** | ENSMUSG00000026411 | chr1:137904796-137931607 | chr1:134717887 - 135889744 | |
| **Zkscan1** | ENSMUSG00000029729 | chr5:138526312-138549050 | chr5:132245840 - 136972814 | |
| **Bik** | ENSMUSG00000016758 | chr15:83357292-83375065 | chr15:81150967 - 85446484 | |

| | | | | |
|---|---|---|---|---|
| **Mup14** | ENSMUSG00000073830 | chr4:60961055-60965051 | chr4:56286549 - 62628686 | + |
| **Dhx40** | ENSMUSG00000018425 | chr11:86582348-86621198 | chr11:78160552 - 93000422 | |
| **Mccc1** | ENSMUSG00000027709 | chr3:35858234-35899600 | chr3:30650923 - 38350217 | |
| **9530068E07Rik** | ENSMUSG00000036275 | chr11:52209930-52222230 | chr11:49789178 - 54407017 | |
| **Mup18** | ENSMUSG00000078674 | chr4:61331211-61335170 | chr4:50569829 - 62263957 | |
| **Glo1** | ENSMUSG00000024026 | chr17:30729811-30749604 | chr17:28832865 - 34004810 | + |
| **Dhrs7** | ENSMUSG00000021094 | chr12:73751343-73765815 | chr12:64596188 - 76093908 | |
| **Rplp0** | ENSMUSG00000067274 | chr5:116009476-116013736 | chr5:113732014 - 120695426 | |
| **Cml5** | ENSMUSG00000079494 | chr6:85767215-85770966 | chr6:84916136 - 87478531 | |
| **Ctse** | ENSMUSG00000004552 | chr1:133534883-133572080 | chr1:130885402 - 137392496 | |
| **Fastkd1** | ENSMUSG00000027086 | chr2:69524872-69551573 | chr2:63020015 - 68902080 | |
| **Cfhr2** | ENSMUSG00000033898 | chr1:141701945-141915481 | chr1:135889744 - 156775182 | + |
| **Cd59a** | ENSMUSG00000032679 | chr2:103935958-103955511 | chr2:101295269 - 112919736 | + |
| **Rps3a** | ENSMUSG00000028081 | chr3:85941862-85946624 | chr3:84573560 - 87186087 | |
| **Rnaset2a** | ENSMUSG00000095687 | chr17:8321456-8340697 | chr17:5764944 - 12772975 | |
| **Zfp945** | ENSMUSG00000059142 | chr17:22983664-23004101 | chr17:16677765 - 23570054 | |
| **Fmo1** | ENSMUSG00000040181 | chr1:164759692-164796679 | chr1:160125359 - 165053877 | |
| **Pop4** | ENSMUSG00000030423 | chr7:39047839-39056367 | chr7:36431337 - 47726947 | |
| **Gm4952** | ENSMUSG00000071633 | chr19:12674506-12702106 | chr19:3250617 - 12675766 | |
| **Pex7** | ENSMUSG00000020003 | chr10:19579735-19627495 | chr10:18992291 - 23321540 | |
| **2810055F11Rik** | ENSMUSG00000019718 | chr12:73174415-73186426 | chr12:71243758 - 74518762 | |
| **Krt23** | ENSMUSG00000006777 | chr11:99339288-99354451 | chr11:96228660 - 98654237 | |
| **Rnf149** | ENSMUSG00000048234 | chr1:39608141-39634250 | chr1:37382716 - 43147962 | |
| **Aldh8a1** | ENSMUSG00000037542 | chr10:21097097-21116391 | chr10:18992291 - 23692494 | |
| **Ifi35** | ENSMUSG00000010358 | chr11:101309721-101320012 | chr11:101084992 - 102590035 | |
| **Vps41** | ENSMUSG00000041236 | chr13:18809161-18958678 | chr13:10959686 - 19993476 | |
| **Cfhr1** | ENSMUSG00000057037 | chr1:141443639-141456835 | chr1:139843091 - 145874225 | + |
| **Hnf1aos1** | ENSMUSG00000086054 | chr5:115418793-115448055 | chr2:117135467 - 121174432 | |
| **Ganc** | ENSMUSG00000062646 | chr2:120229632-120287436 | chr5:114009955 - 116322846 | + |
| **Raet1d** | ENSMUSG00000078452 | chr10:22081700-22093945 | chr10:18992291 - 23433558 | |
| **Rdh9** | ENSMUSG00000056148 | chr10:127196843-127229753 | chr10:124997746 - 129614408 | |
| **Prelid2** | ENSMUSG00000056671 | chr18:42035350-42110848 | chr18:39887772 - 41969216 | |
| **Lyz2** | ENSMUSG00000069516 | chr10:116714390-116719377 | chr10:117418508 - 117499970 | |
| **Zfp672** | ENSMUSG00000049755 | chr11:58128616-58136848 | chr11:52973921 - 54691456 | + |
| **Gbe1** | ENSMUSG00000022707 | chr16:70314194-70569961 | chr16:67445464 - 76724606 | |
| **Alad** | ENSMUSG00000028393 | chr4:62170203-62180952 | chr4:60933569 - 62628686 | |
| **Dpy19l1** | ENSMUSG00000043067 | chr9:24216220-24307584 | chr7:48002471 - 51825053 | |
| **0610005C13Rik** | ENSMUSG00000085214 | chr7:52823165-52830697 | chr9:13879037 - 26203964 | + |
| **Gm4723** | ENSMUSG00000078898 | chr2:175197937-175209702 | chr2:172474381 - 178820577 | |
| **Gm4956** | ENSMUSG00000025936 | chr1:21275327-21288393 | chr1:19809537 - 25632823 | |
| **Mup16** | ENSMUSG00000078675 | chr4:61176624-61180563 | chr4:62263957 - 64029884 | + |
| **Tor1aip2** | ENSMUSG00000050565 | chr1:157882856-157900866 | chr1:153141822 - 161901171 | |
| **Gm14400** | ENSMUSG00000083817 | chr2:177010178-177013668 | chr2:173113613 - 174719530 | |

| | | | | |
|---|---|---|---|---|
| **Rnaset2b** | ENSMUSG00000094724 | chr17:7183209-7202542 | chr17:5764944 - 9038183 | |
| **Arsb** | ENSMUSG00000042082 | chr13:94541634-94712971 | chr13:93099110 - 96407181 | |
| **Ido2** | ENSMUSG00000031549 | chr8:25642364-25686805 | chr8:24904536 - 25400206 | |
| **U2af1** | ENSMUSG00000061613 | chr17:31784027-31795699 | chr17:23904929 - 32075473 | |
| **Rpl3** | ENSMUSG00000060036 | chr15:79908211-79913836 | chr15:77221461 - 81956141 | |
| **Acbd4** | ENSMUSG00000056938 | chr11:102962996-102973514 | chr11:100977097 - 102706300 | |
| **H2-Q2** | ENSMUSG00000091705 | chr17:35479187-35483707 | chr17:26680548 - 33591553 | + |
| **9630013D21Rik** | ENSMUSG00000059027 | chr4:109324134-109330458 | chr4:108857593 - 115924444 | |
| **Gm13152** | ENSMUSG00000078496 | chr4:146866526-146887595 | chr4:140490497 - 143862712 | |
| **Atp5e** | ENSMUSG00000016252 | chr2:174286573-174289606 | chr2:174719530 - 180057625 | |
| **1300010F03Rik** | ENSMUSG00000058997 | chr14:79248985-79602117 | chr14:76127589 - 83519019 | |
| **Ephx2** | ENSMUSG00000022040 | chr14:66703214-66743337 | chr14:59746030 - 69947586 | |
| **Atp9a** | ENSMUSG00000027546 | chr2:168459938-168567909 | chr2:168184899 - 169141273 | + |
| **Ak3** | ENSMUSG00000024782 | chr19:29095323-29122451 | chr19:25336435 - 30239534 | |
| **Iqcc** | ENSMUSG00000040795 | chr4:129292370-129296343 | chr4:128117594 - 128319434 | |
| **Cib1** | ENSMUSG00000030538 | chr7:87372042-87377699 | chr7:83041480 - 83793063 | |
| **Atox1** | ENSMUSG00000018585 | chr11:55260143-55274741 | chr11:54156015 - 58771634 | |
| **Mthfsl** | ENSMUSG00000079427 | chr9:88583443-88614636 | chr9:86522016 - 94131022 | + |
| **Ces2h** | ENSMUSG00000091813 | chr8:107538350-107544310 | chr8:112100634 - 114627710 | |
| **Acad12** | ENSMUSG00000042647 | chr5:122048293-122068947 | chr5:120774344 - 123203687 | + |
| **Josd2** | ENSMUSG00000038695 | chr7:51723350-51727022 | chr7:48002471 - 53769845 | + |
| **9130401M01Rik** | ENSMUSG00000022362 | chr15:57853831-57907980 | chr15:55151291 - 55777903 | |
| **Gas5** | ENSMUSG00000053332 | chr1:162964553-162968670 | chr1:162527085 - 164654736 | + |
| **Nnt** | ENSMUSG00000025453 | chr13:120124255-120197804 | chr13:119378784 - 120116981 | |
| **Mppe1** | ENSMUSG00000062526 | chr18:67385184-67405484 | chr18:63960511 - 68330850 | |
| **H2-K2** | ENSMUSG00000067203 | chr17:34111731-34115733 | chr17:31981493 - 41053604 | + |
| **Mgst3** | ENSMUSG00000026688 | chr1:169302097-169323972 | chr1:169137266 - 174631935 | |
| **Rsph3b** | ENSMUSG00000023806 | chr17:7109030-7153070 | chr17:6102490 - 9315295 | |
| **Vps52** | ENSMUSG00000024319 | chr17:34092757-34103929 | chr17:32134283 - 36234565 | |
| **Gfra1** | ENSMUSG00000025089 | chr19:58310094-58530399 | chr19:57786621 - 58789289 | |
| **Tars2** | ENSMUSG00000028107 | chr3:95543899-95558900 | chr3:97937294 - 105067333 | |
| **Rassf4** | ENSMUSG00000042129 | chr6:116583026-116623854 | chr6:114969395 - 117731966 | |
| **Cyp3a13** | ENSMUSG00000029727 | chr5:138334160-138362847 | chr5:135357302 - 139739343 | |
| **Tpm3** | ENSMUSG00000027940 | chr3:89876571-89904824 | chr3:88125956 - 92375879 | + |
| **Rps29** | ENSMUSG00000034892 | chr12:70258709-70260173 | chr12:59677221 - 71995861 | |
| **Btbd9** | ENSMUSG00000062202 | chr17:30352469-30713394 | chr17:28832865 - 34004810 | |
| **Atxn10** | ENSMUSG00000016541 | chr15:85166768-85294266 | chr15:88347170 - 88973247 | |
| **Tpmt** | ENSMUSG00000021376 | chr13:47120539-47140106 | chr13:45215261 - 52593069 | + |
| **Timd2** | ENSMUSG00000040413 | chr11:46482462-46520563 | chr11:44364024 - 47860405 | + |
| **Gm14327** | ENSMUSG00000074521 | chr2:177631801-177640489 | chr2:178667105 - 178820577 | |
| **Bmp1** | ENSMUSG00000022098 | chr14:70874364-70920067 | chr14:62241300 - 69947586 | |
| **Cisd3** | ENSMUSG00000078695 | chr11:97547140-97549935 | chr11:96717713 - 99410147 | |
| **Aldh16a1** | ENSMUSG00000007833 | chr7:52396054-52409954 | chr7:47774855 - 52606809 | |

| | | | | |
|---|---|---|---|---|
| **Pet112l** | ENSMUSG00000028085 | chr3:85378041-85459544 | chr3:86230898 - 87489032 | |
| **Camk1d** | ENSMUSG00000039145 | chr2:5214503-5635561 | chr2:3046350 - 5376261 | |
| **Sort1** | ENSMUSG00000068747 | chr3:108087009-108164429 | chr3:108797979 - 115203958 | |
| **Psmc5** | ENSMUSG00000020708 | chr11:106117468-106124434 | chr11:101297382 - 106288549 | |
| **Gm14324** | ENSMUSG00000080709 | chr2:177469005-177473462 | chr2:178820577 - 179458127 | |
| **Ugt1a6b** | ENSMUSG00000090145 | chr1:89999827-90115578 | chr1:89753059 - 99119962 | + |
| **Nme7** | ENSMUSG00000026575 | chr1:166243010-166334805 | chr1:161327007 - 162059087 | + |
| **Slc39a2** | ENSMUSG00000072572 | chr14:52512564-52516420 | chr14:48333656 - 52864014 | |
| **Tmem66** | ENSMUSG00000031532 | chr8:35217617-35233899 | chr8:34524120 - 46579264 | |
| **Smyd1** | ENSMUSG00000055027 | chr6:71163934-71272227 | chr6:71680163 - 73284518 | |
| **Fam84b** | ENSMUSG00000072568 | chr15:60650551-60656635 | chr15:62178117 - 63343000 | |
| **Gm16286** | ENSMUSG00000024571 | chr18:80403504-80409480 | chr18:78803726 - 79776136 | + |
| **Fxn** | ENSMUSG00000059363 | chr19:24335943-24355095 | chr19:24024008 - 24849073 | |
| **Ttc38** | ENSMUSG00000035944 | chr15:85662734-85689252 | chr15:86679283 - 87408379 | |
| **Srp54a** | ENSMUSG00000021020 | chr12:56181557-56214680 | chr15:9358458 - 12552201 | |
| **Fyb** | ENSMUSG00000022148 | chr15:6529871-6613321 | chr12:56326785 - 59981234 | |
| **Mup12** | ENSMUSG00000094793 | chr4:60732255-60736198 | chr4:59800888 - 62628686 | + |
| **BC029722** | ENSMUSG00000074649 | chr2:155643466-155645162 | chr2:152091534 - 162469673 | |
| **Mettl6** | ENSMUSG00000021891 | chr14:32291506-32308216 | chr14:31712196 - 32638945 | |
| **1810046J19Rik** | ENSMUSG00000002580 | chr11:98299022-98300305 | chr11:102078561 - 104078038 | |
| **Sfxn2** | ENSMUSG00000025036 | chr19:46647855-46671388 | chr19:44610432 - 44782956 | |
| **Ptprd** | ENSMUSG00000028399 | chr4:75587142-77857865 | chr4:74351764 - 75560945 | |
| **Twf1** | ENSMUSG00000022451 | chr15:94408382-94420320 | chr15:94121629 - 94561654 | |
| **Gm10499** | ENSMUSG00000073403 | chr17:36278703-36282868 | chr17:36883101 - 41053604 | + |
| **Tsen15** | ENSMUSG00000014980 | chr1:154217865-154233818 | chr1:155133452 - 156057381 | |
| **Akr1c19** | ENSMUSG00000071551 | chr13:4227946-4247927 | chr13:3220944 - 6511231 | |
| **Cutc** | ENSMUSG00000025193 | chr19:43827486-43843128 | chr19:40118496 - 40951709 | |
| **Mthfs** | ENSMUSG00000066442 | chr9:89094217-89135064 | chr9:87569635 - 94042526 | |
| **H2-Bl** | ENSMUSG00000073406 | chr17:36201920-36221168 | chr17:32219846 - 40179466 | |
| **Cyp2d12** | ENSMUSG00000096852 | chr15:82385538-82389843 | chr15:84471561 - 86334924 | |
| **Cyp2c44** | ENSMUSG00000025197 | chr19:44079512-44103737 | chr19:40658504 - 40951709 | |
| **Hist1h4i** | ENSMUSG00000060639 | chr13:22132685-22133221 | chr13:20135475 - 23208302 | |
| **Agmat** | ENSMUSG00000040706 | chr4:141302587-141315178 | chr4:141057905 - 142565855 | |
| **Gm5970** | ENSMUSG00000085977 | chr18:60380497-60381712 | chr18:57441750 - 61265879 | |
| **Aen** | ENSMUSG00000030609 | chr7:86040740-86056095 | chr7:86216568 - 86490361 | + |
| **Icam1** | ENSMUSG00000037405 | chr9:20820404-20833241 | chr9:13182953 - 18108766 | |
| **BC026585** | ENSMUSG00000033488 | chr1:159388711-159419191 | chr1:161327007 - 162059087 | |
| **Fam173a** | ENSMUSG00000057411 | chr17:25927453-25929332 | chr17:26680548 - 29365788 | |
| **Gm14305** | ENSMUSG00000078878 | chr2:176493037-176506522 | chr2:178329262 - 178581580 | |
| **Gm10768** | ENSMUSG00000074828 | chr19:43913293-43915218 | chr19:35104083 - 42200247 | |
| **Fam173b** | ENSMUSG00000039065 | chr15:31531607-31550982 | chr15:34086691 - 34700085 | |
| **Mtdh** | ENSMUSG00000022255 | chr15:34012449-34072141 | chr15:33266937 - 34209722 | |
| **Hemk1** | ENSMUSG00000032579 | chr9:107230023-107240681 | chr9:103912182 - 106461499 | |

| | | | | |
|---|---|---|---|---|
| **Aga** | ENSMUSG00000031521 | chr8:54597080-54608776 | chr8:55357356 - 55474478 | |
| **Sympk** | ENSMUSG00000023118 | chr7:19609726-19639967 | chr7:17732740 - 17769986 | |
| **Pccb** | ENSMUSG00000032527 | chr9:100882451-100935317 | chr9:102836554 - 103753663 | |
| **Npc1** | ENSMUSG00000024413 | chr18:12348202-12394909 | chr18:10294491 - 12958657 | |
| **Tbcb** | ENSMUSG00000006095 | chr7:31009150-31017291 | chr7:30068013 - 30342030 | |
| **Cd63** | ENSMUSG00000025351 | chr10:128345975-128349872 | chr10:124997746 - 127910154 | |
| **Snrnp25** | ENSMUSG00000040767 | chr11:32105415-32108984 | chr11:35248737 - 35431622 | |
| **Fam114a2** | ENSMUSG00000020523 | chr11:57296492-57332146 | chr11:56772816 - 57104608 | + |
| **Steap3** | ENSMUSG00000026389 | chr1:122087334-122169282 | chr1:124179367 - 125357830 | |
| **Rhoc** | ENSMUSG00000002233 | chr3:104591929-104597377 | chr3:105422273 - 106995838 | |
| **Trim44** | ENSMUSG00000027189 | chr2:102140276-102247985 | chr2:101511867 - 102765376 | |
| **Zfp949** | ENSMUSG00000032425 | chr9:88442858-88465924 | chr9:87645120 - 88392923 | |
| **Cyp3a25** | ENSMUSG00000029630 | chr5:146788770-146821194 | chr5:145222513 - 147514088 | + |
| **Ttc32** | ENSMUSG00000066637 | chr12:9036803-9043200 | chr12:7608369 - 10569545 | |
| **Tgtp1** | ENSMUSG00000078922 | chr11:48798829-48807674 | chr11:45817537 - 47002325 | |
| **Psma7** | ENSMUSG00000027566 | chr2:179771078-179777138 | chr2:178581580 - 179823855 | |
| **Pex19** | ENSMUSG00000003464 | chr1:174056886-174066624 | chr1:172963069 - 173836444 | |
| **Enpep** | ENSMUSG00000028024 | chr3:128972093-129035638 | chr3:127014677 - 127812337 | |
| **Siae** | ENSMUSG00000001942 | chr9:37421432-37455903 | chr9:40327804 - 40363766 | |
| **Hmga1** | ENSMUSG00000046711 | chr17:27693565-27700619 | chr17:27449729 - 28040946 | |
| **Tmem51** | ENSMUSG00000040616 | chr4:141586907-141640219 | chr4:144378497 - 144928744 | |
| **Swi5** | ENSMUSG00000044627 | chr2:32134336-32143595 | chr2:31941411 - 34714988 | |
| **Nedd4l** | ENSMUSG00000024589 | chr18:65047410-65377480 | chr18:65669450 - 67177732 | |
| **Sarnp** | ENSMUSG00000078427 | chr10:128258827-128314694 | chr10:122443727 - 123707083 | |
| **Trmt2b** | ENSMUSG00000067369 | chr20:130756878-130811523 | chr20:128535461 - 128695239 | |
| **Fam32a** | ENSMUSG00000003039 | chr8:74743629-74747663 | chr18:70047972 - 70244965 | |
| **Ccdc68** | ENSMUSG00000038903 | chr18:70085080-70129138 | chr8:76941137 - 79402559 | |
| **Tmem128** | ENSMUSG00000067365 | chr5:38651424-38660861 | chr5:36737332 - 37813528 | + |
| **1500003O03Rik** | ENSMUSG00000014077 | chr2:119373433-119412763 | chr2:115859084 - 115994093 | |
| **Rpl35a** | ENSMUSG00000060636 | chr16:33056539-33060275 | chr16:36387277 - 37305160 | |
| **Sulf2** | ENSMUSG00000006800 | chr2:165898589-165981163 | chr2:166865009 - 167656617 | |
| **D2hgdh** | ENSMUSG00000073609 | chr1:95721817-95748925 | chr1:93545218 - 93919426 | |
| **Adh6-ps1** | ENSMUSG00000090306 | chr3:138037085-138051255 | chr3:135696847 - 138761310 | |
| **Thnsl2** | ENSMUSG00000054474 | chr6:71078160-71094340 | chr6:74630425 - 76434273 | |
| **Mup2** | ENSMUSG00000078688 | chr4:60080259-60167161 | chr4:59981393 - 61954886 | + |
| **Nsmce2** | ENSMUSG00000059586 | chr15:59205753-59433238 | chr15:43929680 - 55777903 | |
| **C1rb** | ENSMUSG00000098470 | chr6:124462790-124531062 | chr6:118394139 - 120845297 | |
| **1100001G20Rik** | ENSMUSG00000051748 | chr11:83560442-83566144 | chr11:83275936 - 88099515 | |
| **Hook1** | ENSMUSG00000028572 | chr4:95633931-95692104 | chr4:90384356 - 91196614 | |
| **Acads** | ENSMUSG00000029545 | chr5:115560308-115569355 | chr5:115648291 - 116322846 | |
| **Alg1** | ENSMUSG00000039427 | chr16:5233714-5245005 | chr16:3451115 - 3451115 | |
| **Churc1** | ENSMUSG00000090258 | chr12:77866525-77884167 | chr12:76093908 - 80830398 | + |
| **Ilvbl** | ENSMUSG00000032763 | chr10:78037245-78047243 | chr7:13023046 - 16816078 | |

| | | | | |
|---|---|---|---|---|
| **Clptm1** | ENSMUSG00000002981 | chr7:20216930-20250379 | chr10:80277553 - 83776508 | |
| **1110034B05Rik** | ENSMUSG00000048495 | chr1:57445081-57463945 | chr1:58868976 - 59359404 | |
| **Tuba1a** | ENSMUSG00000072235 | chr15:98780272-98783982 | chr15:97987449 - 100348493 | |
| **Hist1h2bl** | ENSMUSG00000094338 | chr13:21807632-21808012 | chr13:22102442 - 25377069 | |
| **Tff3** | ENSMUSG00000024029 | chr17:31262251-31266591 | chr7:51368098 - 51825053 | |
| **Ocel1** | ENSMUSG00000002396 | chr8:73895197-73903260 | chr17:29365788 - 31626873 | |
| **Coq5** | ENSMUSG00000041733 | chr5:115729675-115746981 | chr5:115648291 - 116793513 | |
| **Abcc6** | ENSMUSG00000030834 | chr7:53231750-53285672 | chr8:70997820 - 71556831 | |
| **Pde9a** | ENSMUSG00000041119 | chr17:31523179-31613255 | chr17:27582938 - 28832865 | |
| **Rpl7a** | ENSMUSG00000062647 | chr2:26766284-26768838 | chr2:20233324 - 23363299 | |
| **Acss3** | ENSMUSG00000035948 | chr10:106373220-106560724 | chr10:101832662 - 104599234 | |
| **Pnpt1** | ENSMUSG00000020464 | chr11:29030744-29061828 | chr11:32354761 - 33472055 | |
| **Fam134a** | ENSMUSG00000049339 | chr1:75139360-75144483 | chr1:73851881 - 74132316 | |
| **Tnfsf10** | ENSMUSG00000039304 | chr3:27215950-27241349 | chr3:26448202 - 27787352 | |
| **B4galnt1** | ENSMUSG00000006731 | chr10:126602247-126609385 | chr10:127032881 - 127910154 | |
| **Ercc3** | ENSMUSG00000024382 | chr18:32399954-32429805 | chr18:32937368 - 34093517 | |
| **Hddc3** | ENSMUSG00000030532 | chr7:87488023-87490983 | chr7:85815782 - 86057235 | |
| **Syngr1** | ENSMUSG00000022415 | chr15:79921764-79949931 | chr15:77983701 - 81455222 | |
| **Kmo** | ENSMUSG00000039783 | chr1:177550512-177592247 | chr1:175192149 - 175751076 | + |
| **Sil1** | ENSMUSG00000024357 | chr18:35426054-35658579 | chr18:36845793 - 37673413 | |
| **Jkamp** | ENSMUSG00000005078 | chr12:73186576-73202448 | chr15:52238867 - 54813063 | |
| **Colec10** | ENSMUSG00000038591 | chr15:54242329-54297913 | chr12:71045072 - 71327447 | |
| **Ddx21** | ENSMUSG00000020075 | chr10:62042996-62065046 | chr10:65944206 - 66992234 | |
| **Srp54b** | ENSMUSG00000079108 | chr12:56181483-56290200 | chr3:129316175 - 130169386 | |
| **Cyp2u1** | ENSMUSG00000027983 | chr3:130993409-131006145 | chr12:57292411 - 58167435 | |
| **Eci3** | ENSMUSG00000021416 | chr13:35038483-35055679 | chr13:37544128 - 37602829 | |
| **Rab13** | ENSMUSG00000027935 | chr3:90017617-90030307 | chr3:87369064 - 87489032 | |
| **Gm5834** | ENSMUSG00000085867 | chr1:141836178-141890782 | chr1:139144555 - 139947892 | |
| **Cyp2a22** | ENSMUSG00000091867 | chr7:27716650-27724405 | chr7:28693083 - 28747632 | |
| **5730455O13Rik** | ENSMUSG00000054237 | chr19:38262971-38298628 | chr19:38276072 - 40577851 | |
| **Srd5a3** | ENSMUSG00000029233 | chr5:76569296-76584529 | chr5:75464587 - 76367953 | |
| **Cfhr3** | ENSMUSG00000090623 | chr1:141471762-141524888 | chr1:145976100 - 150856928 | |
| **Gm14325** | ENSMUSG00000095362 | chr2:177566496-177575041 | chr2:172826969 - 172979768 | |
| **Cyp46a1** | ENSMUSG00000021259 | chr12:109572587-109600441 | chr12:109150983 - 110221637 | |
| **Ublcp1** | ENSMUSG00000041231 | chr11:44268073-44284000 | chr20:165220180 - 165475735 | |
| **Pir** | ENSMUSG00000031379 | chr20:160707303-160810943 | chr11:47252882 - 47763140 | |
| **Pctp** | ENSMUSG00000020553 | chr11:89843979-89864208 | chr11:86695966 - 87603851 | |
| **Zbtb8os** | ENSMUSG00000057572 | chr4:129012939-129031791 | chr4:130090374 - 130374753 | |
| **Pebp1** | ENSMUSG00000032959 | chr5:117732660-117737634 | chr5:121209392 - 121422550 | |
| **Dsg2** | ENSMUSG00000044393 | chr18:20716575-20763022 | chr18:21391462 - 22172698 | |
| **Cast** | ENSMUSG00000021585 | chr13:74831691-74945958 | chr13:77005581 - 77081304 | |
| **H2-T10** | ENSMUSG00000079491 | chr17:36252821-36258410 | chr12:113819608 - 114430776 | |

| | | | | |
|---|---|---|---|---|
| **2810002N01Rik** | ENSMUSG00000037787 | chr12:112951472-112993191 | chr17:36047232 - 37823197 | |
| **Pigr** | ENSMUSG00000026417 | chr1:132723261-132748826 | chr1:127042332 - 128332495 | |
| **Ugt3a1** | ENSMUSG00000072664 | chr15:9203074-9244787 | chr15:11689950 - 11786564 | |
| **Gm8909** | ENSMUSG00000073402 | chr17:36301388-36305482 | chr17:40593701 - 41133516 | |
| **Rhot2** | ENSMUSG00000025733 | chr17:25975402-25981796 | chr17:27582938 - 28082777 | |
| **Oxnad1** | ENSMUSG00000021906 | chr14:32898560-32916388 | chr14:32638945 - 33480782 | |
| **Wbscr27** | ENSMUSG00000040557 | chr5:135408238-135418507 | chr6:123129728 - 123951869 | |
| **Gm10319** | ENSMUSG00000071204 | chr6:122086646-122102034 | chr5:135656531 - 136308711 | |
| **Gm14326** | ENSMUSG00000078862 | chr2:177670698-177692002 | chr2:173679868 - 174719530 | |
| **C920025E04Rik** | ENSMUSG00000073405 | chr17:36245531-36248633 | chr17:32219846 - 32532642 | |
| **Ceacam1** | ENSMUSG00000074272 | chr7:26246721-26262644 | chr7:25938623 - 26080030 | |
| **Acyp1** | ENSMUSG00000008822 | chr12:86613348-86629388 | chr12:88200646 - 88322904 | |
| **Iigp1** | ENSMUSG00000054072 | chr18:60535683-60552281 | chr18:58671982 - 59474939 | |
| **Cst3** | ENSMUSG00000027447 | chr2:148697458-148701428 | chr2:146734052 - 147092254 | |
| **Dmgdh** | ENSMUSG00000042102 | chr13:94444388-94522786 | chr13:96175325 - 96407181 | |
| **Slco1a1** | ENSMUSG00000041698 | chr6:141855803-141895483 | chr6:142550412 - 143061552 | |
| **Hn1l** | ENSMUSG00000024165 | chr17:25079101-25097581 | chr17:18081650 - 21525383 | |
| **Top1mt** | ENSMUSG00000000934 | chr15:75487465-75509230 | chr15:73873324 - 73956847 | |
| **Fam176a** | ENSMUSG00000035104 | chr6:81991037-82043093 | chr6:85419119 - 86219915 | |
| **Tbrg4** | ENSMUSG00000000384 | chr11:6515601-6526070 | chr11:6566421 - 7497501 | |
| **Cyp2d41-ps** | ENSMUSG00000058613 | chr15:82608361-82612540 | chr15:80218764 - 81051691 | |
| **Cobl** | ENSMUSG00000020173 | chr11:12136611-12364963 | chr11:11775990 - 12418467 | |
| **Wdr83** | ENSMUSG00000005150 | chr8:87598934-87605205 | chr17:31313309 - 32075473 | |
| **H2-T23** | ENSMUSG00000067212 | chr17:36166718-36169800 | chr15:62178117 - 62265191 | |
| **E430025E21Rik** | ENSMUSG00000022350 | chr15:59163553-59205722 | chr8:88491121 - 91129945 | |
| **Gm14421** | ENSMUSG00000083111 | chr2:176838221-176841543 | chr2:179033792 - 179133740 | |
| **Ppie** | ENSMUSG00000028651 | chr4:122804358-122817194 | chr4:127491886 - 127646156 | |
| **Mgmt** | ENSMUSG00000054612 | chr7:144086294-144319870 | chr7:142321560 - 144278127 | |
| **Prkcz** | ENSMUSG00000029053 | chr4:154634238-154735470 | chr4:154337446 - 154497501 | |
| **Anapc5** | ENSMUSG00000029472 | chr5:123237478-123271348 | chr5:126043804 - 126124155 | |
| **Hist1h2bk** | ENSMUSG00000062727 | chr13:22127690-22128168 | chr13:21469813 - 22102442 | |
| **Gm13305** | ENSMUSG00000073876 | chr4:42029565-42039326 | chr4:40452083 - 40936506 | |
| **Spg21** | ENSMUSG00000032388 | chr9:65308744-65336277 | chr7:53502241 - 53769845 | |
| **Raet1c** | ENSMUSG00000053219 | chr10:21893327-21903720 | chr10:22318517 - 23185062 | |
| **Gm15545** | ENSMUSG00000087138 | chr7:52242270-52249967 | chr9:64763234 - 65386738 | |
| **Pkig** | ENSMUSG00000035268 | chr2:163484122-163551894 | chr2:178329262 - 178423190 | |
| **Gm4724** | ENSMUSG00000078897 | chr2:175247801-175261308 | chr2:162539490 - 162618549 | |
| **Rmnd1** | ENSMUSG00000019763 | chr10:5914153-5944626 | chr10:8739506 - 9159166 | |
| **Igsf11** | ENSMUSG00000022790 | chr16:38901584-39027272 | chr16:37075355 - 37305160 | |
| **Rab4a** | ENSMUSG00000019478 | chr8:126329885-126359187 | chr8:125239089 - 125446525 | |
| **H2-Ke2** | ENSMUSG00000024309 | chr17:34075766-34077288 | chr4:93276507 - 94618325 | |
| **Cyp2j5** | ENSMUSG00000052520 | chr4:96295464-96330845 | chr17:33839211 - 34542617 | |
| **Ngrn** | ENSMUSG00000047084 | chr7:87406101-87410264 | chr7:82936839 - 83263051 | |

| | | | | |
|---|---|---|---|---|
| **Srp54c** | ENSMUSG00000073079 | chr12:56331155-56377912 | chr12:60468769 - 61291973 | |
| **Fam55b** | ENSMUSG00000032028 | chr9:48126089-48161537 | chr9:49404442 - 49601221 | |
| **Abhd3** | ENSMUSG00000002475 | chr18:10644409-10706769 | chr18:5607870 - 6596284 | |
| **1110031I02Rik** | ENSMUSG00000025169 | chr11:121038905-121066025 | chr11:118563976 - 118676908 | |
| **F830016B08Rik** | ENSMUSG00000090942 | chr18:60453034-60462670 | chr18:63831810 - 64160832 | |
| **Brap** | ENSMUSG00000029458 | chr5:122110572-122137257 | chr5:122321928 - 122959060 | |
| **Sel1l3** | ENSMUSG00000029189 | chr5:53498323-53604691 | chr5:53749401 - 53831096 | |
| **Gm16363** | ENSMUSG00000085513 | chr2:177326386-177327262 | chr2:173113613 - 173333432 | |
| **Ndufs5** | ENSMUSG00000028648 | chr4:123389953-123395445 | chr4:125979990 - 126162299 | |
| **Frrs1** | ENSMUSG00000033386 | chr3:116581145-116606668 | chr3:111987339 - 114102206 | |
| **Glrx2** | ENSMUSG00000018196 | chr1:145586159-145596806 | chr1:145874225 - 146136935 | |
| **H2-T22** | ENSMUSG00000056116 | chr17:36174073-36179692 | chr17:35604221 - 36047232 | |
| **Cox16** | ENSMUSG00000091803 | chr12:82460013-82586114 | chr12:82787731 - 83122752 | |
| **Plekhb2** | ENSMUSG00000026123 | chr1:34906804-34936425 | chr1:30403172 - 31692631 | |
| **Dnajc8** | ENSMUSG00000054405 | chr4:132091465-132109657 | chr4:132517235 - 133530440 | |
| **Ttc39c** | ENSMUSG00000024424 | chr18:12758435-12895559 | chr18:15241946 - 15486956 | |
| **Gstz1** | ENSMUSG00000021033 | chr12:88488668-88505673 | chr12:90750572 - 90850469 | |
| **Cyb5r4** | ENSMUSG00000032872 | chr9:86916849-86972609 | chr9:86419856 - 87123415 | |
| **St3gal1** | ENSMUSG00000013846 | chr15:66934437-66945922 | chr15:64806921 - 65641372 | |
| **Tpd52** | ENSMUSG00000027506 | chr3:8928626-9004723 | chr3:10458755 - 10606549 | |
| **Nat8** | ENSMUSG00000030004 | chr6:85780382-85782076 | chr6:85262721 - 85493102 | |
| **Cyp4f17** | ENSMUSG00000091586 | chr17:32643407-32665839 | chr17:28200597 - 28832865 | |
| **2010305A19Rik** | ENSMUSG00000048351 | chr4:108000745-108014149 | chr4:106639513 - 106967190 | |
| **Mup5** | ENSMUSG00000058523 | chr4:61492353-61496267 | chr4:62952000 - 63947895 | |
| **Nudcd2** | ENSMUSG00000020328 | chr11:40547169-40553548 | chr11:38705716 - 38870559 | |
| **S100a13** | ENSMUSG00000042312 | chr3:90318357-90328503 | chr3:90523021 - 92375879 | |
| **Mnd1** | ENSMUSG00000033752 | chr3:83891856-83959708 | chr3:84042371 - 84105429 | |
| **Slc27a5** | ENSMUSG00000030382 | chr7:13573695-13583541 | chr7:12686189 - 12867799 | |
| **Serpina1a** | ENSMUSG00000066366 | chr12:105091799-105104137 | chr12:104830971 - 104919487 | |
| **4833420G17Rik** | ENSMUSG00000062822 | chr13:120251566-120274924 | chr13:119442736 - 119522520 | |
| **Serpine2** | ENSMUSG00000026249 | chr1:79790772-79855271 | chr1:82355538 - 83263723 | |
| **Rap2a** | ENSMUSG00000051615 | chr14:120877666-120906416 | chr14:120888799 - 121011608 | |
| **Mup17** | ENSMUSG00000096688 | chr4:61252961-61256903 | chr4:62703129 - 62952000 | |
| **Galk2** | ENSMUSG00000027207 | chr2:125684845-125810035 | chr2:126817478 - 127285668 | |
| **Plscr2** | ENSMUSG00000032372 | chr9:92170440-92192573 | chr14:47590381 - 47757281 | |
| **Mapk1ip1l** | ENSMUSG00000021840 | chr14:47917966-47948460 | chr9:87473903 - 87569635 | |
| **Rsph3a** | ENSMUSG00000073471 | chr17:8138518-8172689 | chr3:105067333 - 105195661 | |
| **Gstm6** | ENSMUSG00000068762 | chr3:107741765-107746667 | chr17:15328351 - 15407471 | |
| **9030025P20Rik** | ENSMUSG00000079709 | chr17:15115829-15127768 | chr17:11420026 - 11598843 | |
| **Gtf3a** | ENSMUSG00000016503 | chr5:147760233-147767190 | chr5:148760942 - 149413154 | |
| **Ces1g** | ENSMUSG00000057074 | chr8:95826268-95861207 | chr8:99063260 - 99362813 | |
| **Rbfa** | ENSMUSG00000024570 | chr18:80389004-80397397 | chr18:78880086 - 79065141 | |

| | | | | |
|---|---|---|---|---|
| **Pigp** | ENSMUSG00000022940 | chr16:94580370-94592622 | chr16:94079235 - 94454536 | |
| **Zfp68** | ENSMUSG00000058291 | chr5:139045844-139060989 | chr7:106498296 - 106611242 | |
| **1810020D17Rik** | ENSMUSG00000035642 | chr7:104698844-104728007 | chr5:138303964 - 139201081 | |
| **Oasl1** | ENSMUSG00000041827 | chr5:115373249-115387924 | chr5:116889885 - 116986242 | |
| **2510006D16Rik** | ENSMUSG00000028797 | chr4:129277912-129301303 | chr4:128418274 - 128505791 | |
| **Rpl27a** | ENSMUSG00000046364 | chr7:116662661-116665881 | chr7:112298484 - 112483463 | |
| **Pxn** | ENSMUSG00000029528 | chr5:115956685-116005996 | chr5:116049346 - 116193364 | |
| **Selenbp2** | ENSMUSG00000068877 | chr3:94497478-94508335 | chr3:90170556 - 90301424 | |
| **Snap47** | ENSMUSG00000009894 | chr11:59220636-59264688 | chr11:59342773 - 59436822 | |
| **2210010C17Rik** | ENSMUSG00000040498 | chr7:20522654-20536092 | chr7:25192586 - 25335537 | |
| **Gbp11** | ENSMUSG00000092021 | chr5:105755499-105772948 | chr5:105285093 - 105513638 | |
| **Tmem125** | ENSMUSG00000050854 | chr4:118213546-118216649 | chr4:116498165 - 116616203 | |
| **Acsm5** | ENSMUSG00000030972 | chr7:126669818-126687307 | chr7:130938204 - 131532412 | |
| **Gm14420** | ENSMUSG00000078866 | chr2:177249438-177262834 | chr2:179077649 - 179133740 | |
| **Accn5** | ENSMUSG00000028008 | chr3:81786212-81825155 | chr3:82780632 - 82959559 | |
| **Zcchc9** | ENSMUSG00000021621 | chr13:91936138-91947301 | chr13:91865145 - 91965826 | |
| **Hdhd2** | ENSMUSG00000025421 | chr18:77168865-77211641 | chr18:76819460 - 76893898 | |
| **Numb** | ENSMUSG00000021224 | chr12:85134984-85262884 | chr12:88200646 - 88252032 | |
| **D1Bwg0212e** | ENSMUSG00000003135 | chr1:39591837-39603726 | chr8:123951818 - 124054881 | |
| **2810004N23Rik** | ENSMUSG00000031984 | chr8:127363255-127386929 | chr1:37651077 - 37747157 | |
| **Mup1** | ENSMUSG00000078683 | chr4:60510884-60514832 | chr9:109234033 - 109461562 | |
| **Hyal1** | ENSMUSG00000010051 | chr9:107479258-107485174 | chr4:60933569 - 61881691 | |
| **Gm6710** | ENSMUSG00000078887 | chr2:175868351-175880380 | chr2:178513997 - 178581580 | |
| **Txnl4a** | ENSMUSG00000057130 | chr18:80403534-80420272 | chr18:79010972 - 79065141 | |
| **Vkorc1l1** | ENSMUSG00000066735 | chr5:130417979-130460615 | chr5:127894444 - 127957589 | |
| **Gm7120** | ENSMUSG00000074634 | chr13:120276748-120284312 | chr13:119108936 - 119187691 | |
| **Akr1c13** | ENSMUSG00000021213 | chr13:4190430-4204842 | chr13:3220944 - 3220944 | |
| **Nudt19** | ENSMUSG00000034875 | chr7:36332204-36341323 | chr7:37141541 - 37086470 | |
| **Clybl** | ENSMUSG00000025545 | chr14:122580916-122801454 | chr14:122415589 - 122275768 | |

**Table S4. Predicted isoform-ratio eQTLs in liver.** Shown are 243 gene symbols (column 1), their ensemble identifiers (column 2), genomic positions (column 3), and the genomic positions of their top ranking proximal isoform-ratio eQTLs (column 4). Column 5 indicates whether the gene is also significantly associated with a total-expression eQTL, as detailed in **Table S3**.

| Isoform-ratio trait | | | Isoform-ratio eQTL interval | Total-expression eQTL |
|---|---|---|---|---|
| **Symbol** | **Ensembl identifier** | **Genomic position** | | |
| **H2-K1** | ENSMUSG00000061232 | chr17:34132962-34137278 | chr17:33696256 - 41264486 | |
| **Rpl29** | ENSMUSG00000048758 | chr9:106331785-106333899 | chr9:103912182 - 113029682 | + |
| **Rpl21** | ENSMUSG00000041453 | chr5:147644466-147648608 | chr5:141365337 - 152052722 | + |
| **6530401N04Rik** | ENSMUSG00000020956 | chr12:53089299-53107488 | chr12:36564946 - 55557209 | + |
| **Gcap14** | ENSMUSG00000058690 | chr14:37688122-37781950 | chr14:32360414 - 48125148 | |
| **Apoa2** | ENSMUSG00000005681 | chr1:173155185-173156510 | chr1:172174211 - 178009435 | |
| **Hsd3b2** | ENSMUSG00000063730 | chr3:98513178-98528499 | chr3:90170556 - 121682115 | |
| **H2-Q2** | ENSMUSG00000091705 | chr17:35479187-35483707 | chr17:26680548 - 44813750 | + |
| **Mup2** | ENSMUSG00000078688 | chr4:60080259-60167161 | chr4:51674433 - 62549879 | + |
| **Mki67ip** | ENSMUSG00000026377 | chr1:120218416-120230399 | chr1:108999635 - 134466386 | |
| **Vps28** | ENSMUSG00000062381 | chr15:76452518-76456457 | chr15:65156561 - 86334924 | |
| **Shb** | ENSMUSG00000044813 | chr4:45436150-45545342 | chr4:36170536 - 46266471 | |
| **Uqcr10** | ENSMUSG00000059534 | chr11:4601976-4604345 | chr11:3277587 - 11627495 | |
| **Foxo3** | ENSMUSG00000048756 | chr10:41901647-41996561 | chr10:27920057 - 46222885 | |
| **Mup1** | ENSMUSG00000078683 | chr4:60510884-60514832 | chr4:57030717 - 64029884 | + |
| **Churc1** | ENSMUSG00000090258 | chr12:77866525-77884167 | chr12:76015302 - 83122752 | + |
| **Tpm3** | ENSMUSG00000027940 | chr3:89876571-89904824 | chr3:87773377 - 93283619 | + |
| **Pxmp4** | ENSMUSG00000000876 | chr2:154411494-154429444 | chr2:150446861 - 158740971 | |
| **Gas5** | ENSMUSG00000053332 | chr1:162964553-162968670 | chr1:160670702 - 167827635 | + |
| **Nr2f6** | ENSMUSG00000002393 | chr8:73898022-73905859 | chr8:50980992 - 76089799 | |
| **Nr1i3** | ENSMUSG00000005677 | chr1:173144101-173150832 | chr4:32428283 - 38124758 | |
| **1810030N24Rik** | ENSMUSG00000028295 | chr4:34715913-34725672 | chr1:172174211 - 176903126 | |
| **Mbd1** | ENSMUSG00000024561 | chr18:74427926-74442339 | chr18:73164943 - 78880086 | |
| **Qdpr** | ENSMUSG00000015806 | chr5:45825271-45841474 | chr5:41119435 - 46916453 | |
| **Ifi27l1** | ENSMUSG00000064215 | chr12:104672421-104678449 | chr12:103862248 - 108419338 | |
| **Slc35a1** | ENSMUSG00000028293 | chr4:34610506-34634687 | chr4:32428283 - 36531816 | |
| **Nol12** | ENSMUSG00000033099 | chr15:78765363-78774064 | chr15:77221461 - 86900034 | |
| **Glo1** | ENSMUSG00000024026 | chr17:30729811-30749604 | chr17:26633727 - 33696256 | + |
| **Slc17a3** | ENSMUSG00000036083 | chr13:23931303-23952583 | chr13:19993476 - 26089173 | |
| **Fxc1** | ENSMUSG00000089847 | chr7:112788570-112804262 | chr7:116005580 - 119226564 | |
| **Dctn4** | ENSMUSG00000024603 | chr18:60685868-60718416 | chr18:52905351 - 59555613 | |
| **Mrap** | ENSMUSG00000039956 | chr16:90738569-90750030 | chr16:90526174 - 97132278 | |
| **Ubb** | ENSMUSG00000019505 | chr11:62364673-62366715 | chr11:58771634 - 62822705 | |
| **Prg4** | ENSMUSG00000006014 | chr1:152296542-152313295 | chr17:32075473 - 49104468 | |
| **Gm10499** | ENSMUSG00000073403 | chr17:36278703-36282868 | chr1:135334922 - 156165867 | + |

| | | | | |
|---|---|---|---|---|
| **Cd59a** | ENSMUSG00000032679 | chr2:103935958-103955511 | chr2:101420603 - 106279697 | + |
| **Josd2** | ENSMUSG00000038695 | chr7:51723350-51727022 | chr7:47726947 - 53584602 | + |
| **H2-Q4** | ENSMUSG00000035929 | chr17:35516562-35522235 | chr17:32219846 - 41264486 | |
| **Pqlc1** | ENSMUSG00000034006 | chr18:80450031-80489464 | chr18:77531636 - 81587895 | |
| **Mrpl55** | ENSMUSG00000036860 | chr11:59015988-59019636 | chr11:58771634 - 67636607 | |
| **RP23-381B19.7** | ENSMUSG00000093483 | chr11:83105201-83108134 | chr11:81945493 - 93000422 | + |
| **Timm9** | ENSMUSG00000021079 | chr12:72224160-72237662 | chr12:65056299 - 74687060 | |
| **Agphd1** | ENSMUSG00000035878 | chr9:54765090-54797731 | chr9:49846132 - 58199303 | |
| **Nme7** | ENSMUSG00000026575 | chr1:166243010-166334805 | chr1:158184453 - 169750862 | + |
| **Cyp3a25** | ENSMUSG00000029630 | chr5:146788770-146821194 | chr5:145152196 - 148390455 | + |
| **Gm7120** | ENSMUSG00000074634 | chr13:120276748-120284312 | chr13:118816801 - 120116981 | + |
| **Ugt1a6a** | ENSMUSG00000054545 | chr1:89999827-90116577 | chr1:89636728 - 101912869 | |
| **Arsg** | ENSMUSG00000020604 | chr11:109334688-109434644 | chr11:107799738 - 111598527 | |
| **Mvk** | ENSMUSG00000041939 | chr5:114894278-114910600 | chr5:114009955 - 120774344 | |
| **Snx24** | ENSMUSG00000024535 | chr18:53405316-53550477 | chr6:144468689 - 146236620 | |
| **Lyrm5** | ENSMUSG00000040370 | chr6:145159654-145165459 | chr18:43474200 - 54635554 | |
| **Itih1** | ENSMUSG00000006529 | chr14:31742366-31756475 | chr14:27732837 - 33480782 | |
| **Ganc** | ENSMUSG00000062646 | chr2:120229632-120287436 | chr2:120175083 - 128189764 | + |
| **Abhd1** | ENSMUSG00000006638 | chr5:31252439-31257464 | chr5:30282706 - 33446960 | |
| **Dpys** | ENSMUSG00000022304 | chr15:39600031-39689016 | chr15:38781717 - 42160335 | |
| **Trim12c** | ENSMUSG00000057143 | chr7:111487268-111501876 | chr7:116311909 - 119108602 | |
| **Gm4788** | ENSMUSG00000070594 | chr1:141594212-141677816 | chr1:137571698 - 139144555 | |
| **Adhfe1** | ENSMUSG00000025911 | chr1:9538029-9568051 | chr1:6084735 - 16834035 | |
| **1600014C10Rik** | ENSMUSG00000054676 | chr7:38968236-38982582 | chr7:38626755 - 47606066 | |
| **Mup12** | ENSMUSG00000094793 | chr4:60732255-60736198 | chr4:55261729 - 63321340 | + |
| **Prr13** | ENSMUSG00000023048 | chr15:102289459-102293237 | chr15:102056859 - 103031732 | |
| **Cops6** | ENSMUSG00000019494 | chr5:138602299-138605874 | chr5:139008481 - 141365337 | |
| **Echdc1** | ENSMUSG00000019883 | chr10:29032972-29066467 | chr10:26226262 - 26336604 | |
| **Mup14** | ENSMUSG00000073830 | chr4:60961055-60965051 | chr4:51843614 - 62549879 | + |
| **Eif4g2** | ENSMUSG00000005610 | chr7:118211499-118226544 | chr7:115518460 - 116412535 | |
| **Gatad1** | ENSMUSG00000007415 | chr5:3632932-3647934 | chr5:3230193 - 15543723 | |
| **Masp2** | ENSMUSG00000028979 | chr4:147976663-147989608 | chr4:142312877 - 144928744 | |
| **Gsta2** | ENSMUSG00000057933 | chr9:78178825-78203595 | chr9:74596134 - 87645120 | |
| **Creg1** | ENSMUSG00000040713 | chr1:167693877-167705439 | chr1:164792505 - 165053877 | |
| **Glyctk** | ENSMUSG00000020258 | chr9:106055188-106060469 | chr9:108409204 - 110507707 | |
| **Ngef** | ENSMUSG00000026259 | chr1:89373409-89470445 | chr1:88974625 - 92401117 | |
| **Ccbl2** | ENSMUSG00000040213 | chr3:142364015-142407874 | chr3:138761310 - 144815433 | |
| **Tor1aip1** | ENSMUSG00000026466 | chr1:157851729-157883610 | chr1:156775182 - 158059865 | |
| **Slc25a42** | ENSMUSG00000002346 | chr8:72708239-72736204 | chr8:70997820 - 74364169 | |
| **Ugt1a6b** | ENSMUSG00000090145 | chr1:89999827-90115578 | chr1:88974625 - 91699637 | + |
| **Anp32e** | ENSMUSG00000015749 | chr3:95733169-95751313 | chr3:96706017 - 97638598 | |

| | | | | |
|---|---|---|---|---|
| **Ankrd33b** | ENSMUSG00000022237 | chr15:31221234-31297514 | chr15:30367069 - 34700085 | |
| **Spp1** | ENSMUSG00000029304 | chr5:104864137-104870069 | chr5:100739111 - 109031314 | |
| **Mthfsl** | ENSMUSG00000079427 | chr9:88583443-88614636 | chr9:87218402 - 94328348 | + |
| **Mettl21a** | ENSMUSG00000025956 | chr1:64653047-64663816 | chr1:61626676 - 71075258 | |
| **Hint3** | ENSMUSG00000019791 | chr10:30327947-30338285 | chr10:27594228 - 41368505 | |
| **Mpg** | ENSMUSG00000020287 | chr11:32126505-32132700 | chr11:31310649 - 31765528 | |
| **Acad12** | ENSMUSG00000042647 | chr5:122048293-122068947 | chr5:121512914 - 123695117 | + |
| **Serpina3g** | ENSMUSG00000041481 | chr12:105474455-105480149 | chr12:108048615 - 109150983 | |
| **Zfp672** | ENSMUSG00000049755 | chr11:58128616-58136848 | chr11:53568444 - 55970063 | + |
| **Acox2** | ENSMUSG00000021751 | chr14:9058025-9091867 | chr14:5958958 - 13070109 | |
| **2210015D19Rik** | ENSMUSG00000083844 | chr11:5662132-5684713 | chr11:4690979 - 10361203 | |
| **Slc6a13** | ENSMUSG00000030108 | chr6:121250245-121287751 | chr6:116721425 - 121028700 | |
| **Calcoco1** | ENSMUSG00000023055 | chr15:102537210-102552609 | chr15:95717110 - 97902919 | |
| **Timd2** | ENSMUSG00000040413 | chr11:46482462-46520563 | chr11:45086945 - 49043144 | + |
| **Kmo** | ENSMUSG00000039783 | chr1:177550512-177592247 | chr1:175448899 - 183127455 | + |
| **Nudt1** | ENSMUSG00000036639 | chr5:140807814-140814091 | chr5:134927477 - 141365337 | |
| **Aen** | ENSMUSG00000030609 | chr7:86040740-86056095 | chr7:86397253 - 86783593 | + |
| **Mtfmt** | ENSMUSG00000059183 | chr9:65283589-65300861 | chr9:65328543 - 68509840 | |
| **Cyp2c40** | ENSMUSG00000025004 | chr19:39841561-39887304 | chr19:40466136 - 40728030 | |
| **Gm16286** | ENSMUSG00000024571 | chr18:80403504-80409480 | chr18:79868185 - 81414476 | + |
| **Thyn1** | ENSMUSG00000035443 | chr9:26807262-26814915 | chr9:21613279 - 23988491 | |
| **Acaa1a** | ENSMUSG00000036138 | chr9:119248794-119259417 | chr9:119632778 - 122144220 | |
| **Ces3a** | ENSMUSG00000069922 | chr8:107572499-107582314 | chr8:102596452 - 106583721 | |
| **Pih1d1** | ENSMUSG00000003423 | chr7:52409673-52415439 | chr7:48002471 - 51963704 | |
| **Glrx2** | ENSMUSG00000018196 | chr1:145586159-145596806 | chr1:140315338 - 145874225 | + |
| **Ttc39b** | ENSMUSG00000038172 | chr4:82866204-82970159 | chr4:77800490 - 82942547 | |
| **Phldb2** | ENSMUSG00000033149 | chr16:45746346-45953711 | chr16:48719143 - 66177584 | |
| **Mtap7** | ENSMUSG00000019996 | chr10:19868277-20001393 | chr10:18060806 - 19086406 | |
| **Apobec1** | ENSMUSG00000040613 | chr6:122527810-122552462 | chr6:126059914 - 127554876 | |
| **Ubc** | ENSMUSG00000008348 | chr5:125866335-125870572 | chr5:121667878 - 127060447 | |
| **Sc5d** | ENSMUSG00000032018 | chr9:42062260-42072383 | chr9:41639078 - 42317393 | |
| **Zscan21** | ENSMUSG00000037017 | chr5:138558131-138575493 | chr5:135115239 - 138303964 | |
| **Gm8909** | ENSMUSG00000073402 | chr17:36301388-36305482 | chr17:26680548 - 32134283 | + |
| **1700001C19Rik** | ENSMUSG00000047150 | chr17:47549683-47574325 | chr17:44407282 - 47565243 | |
| **Cd55** | ENSMUSG00000026399 | chr1:132335604-132359321 | chr1:130086065 - 130382461 | |
| **Ttll12** | ENSMUSG00000016757 | chr15:83405549-83425587 | chr15:82800868 - 83770961 | |
| **Slc35e1** | ENSMUSG00000019731 | chr8:75001895-75016513 | chr8:77148392 - 78866020 | |
| **Cfhr1** | ENSMUSG00000057037 | chr1:141443639-141456835 | chr1:137392496 - 140315338 | + |
| **Prorsd1** | ENSMUSG00000032673 | chr11:29411757-29415033 | chr11:29934542 - 31420627 | |
| **Nit1** | ENSMUSG00000013997 | chr1:173268139-173275777 | chr1:168178002 - 173836444 | |
| **H2-D1** | ENSMUSG00000073411 | chr17:35399675-35404444 | chr17:34642923 - 41264486 | |

| | | | | |
|---|---|---|---|---|
| **Tpmt** | ENSMUSG00000021376 | chr13:47120539-47140106 | chr13:49978779 - 51831996 | + |
| **Commd5** | ENSMUSG00000055041 | chr15:76730340-76731735 | chr15:76022303 - 77511010 | |
| **Hmgn2** | ENSMUSG00000003038 | chr4:133520653-133524565 | chr4:132435803 - 133999708 | |
| **Atp9a** | ENSMUSG00000027546 | chr2:168459938-168567909 | chr2:168184899 - 171243709 | + |
| **Fbxo6** | ENSMUSG00000055401 | chr4:147519825-147526249 | chr4:149254022 - 149330421 | |
| **Vwa5a** | ENSMUSG00000023186 | chr9:38525853-38550922 | chr9:36885503 - 37959805 | |
| **2310001H17Rik** | ENSMUSG00000097354 | chr6:129158430-129188000 | chr6:128552862 - 129021548 | |
| **Rpn2** | ENSMUSG00000027642 | chr2:157104753-157152055 | chr2:154972094 - 158740971 | |
| **Itgb1bp1** | ENSMUSG00000062352 | chr12:21246686-21292098 | chr12:17297580 - 25481963 | |
| **H2-K2** | ENSMUSG00000067203 | chr17:34111731-34115733 | chr17:36047232 - 41133516 | + |
| **Mrps23** | ENSMUSG00000023723 | chr11:88017890-88025009 | chr11:90361138 - 94477781 | |
| **Lbp** | ENSMUSG00000016024 | chr2:158132229-158158588 | chr2:153804191 - 159415169 | |
| **Ddah1** | ENSMUSG00000028194 | chr3:145421639-145557241 | chr3:144235126 - 147918027 | |
| **Usf1** | ENSMUSG00000026641 | chr1:173341444-173349273 | chr1:170263553 - 171567355 | |
| **Fam114a2** | ENSMUSG00000020523 | chr11:57296492-57332146 | chr11:51857731 - 57286381 | + |
| **Alg6** | ENSMUSG00000073792 | chr4:99382355-99430151 | chr4:96160448 - 99642114 | |
| **Faf2** | ENSMUSG00000025873 | chr13:54723145-54765429 | chr13:57740157 - 59623286 | |
| **Sfr1** | ENSMUSG00000025066 | chr19:47806246-47810078 | chr19:45632134 - 48048757 | |
| **Fgf1** | ENSMUSG00000036585 | chr18:38998327-39089058 | chr18:38200091 - 39987692 | |
| **Echdc3** | ENSMUSG00000039063 | chr2:6109511-6134079 | chr2:5292024 - 5758389 | |
| **Prodh2** | ENSMUSG00000036892 | chr7:31278641-31298421 | chr7:31704657 - 34621203 | |
| **App** | ENSMUSG00000022892 | chr16:84954685-85173952 | chr16:86503354 - 87950231 | |
| **Ccl9** | ENSMUSG00000019122 | chr11:83386421-83392138 | chr11:81251449 - 81887279 | |
| **Lyrm2** | ENSMUSG00000045854 | chr4:32887228-32888534 | chr4:28543236 - 28683004 | |
| **Atp5sl** | ENSMUSG00000057229 | chr7:26404433-26410570 | chr7:25192586 - 25938623 | |
| **Cnot7** | ENSMUSG00000031601 | chr8:41577894-41601201 | chr8:35673393 - 39952821 | |
| **2310045N01Rik** | ENSMUSG00000002345 | chr8:72663398-72691387 | chr8:70997820 - 73879588 | |
| **Tmem128** | ENSMUSG00000067365 | chr5:38651424-38660861 | chr5:39164410 - 39444524 | + |
| **Sdhc** | ENSMUSG00000058076 | chr1:173057296-173080734 | chr1:172825382 - 173057579 | |
| **Ublcp1** | ENSMUSG00000041231 | chr11:44268073-44284000 | chr11:43991141 - 44500887 | + |
| **Sec22b** | ENSMUSG00000027879 | chr3:97705113-97727199 | chr3:96120832 - 96418053 | |
| **Hfe** | ENSMUSG00000006611 | chr13:23793903-23802723 | chr13:20208147 - 23042409 | |
| **Gnl2** | ENSMUSG00000028869 | chr4:124693829-124732624 | chr16:8667124 - 10746532 | |
| **Clec16a** | ENSMUSG00000068663 | chr16:10545457-10744971 | chr4:123543905 - 124034675 | |
| **Ahcy** | ENSMUSG00000027597 | chr2:154885046-154900233 | chr2:158960764 - 161046235 | |
| **Eif2b4** | ENSMUSG00000029145 | chr5:31489931-31495803 | chr5:29356890 - 31080804 | |
| **AI182371** | ENSMUSG00000035875 | chr2:34939566-34957063 | chr2:30440952 - 34714988 | |
| **Gm16573** | ENSMUSG00000090038 | chr14:57226098-57239350 | chr14:57822416 - 58490911 | |
| **1810058I24Rik** | ENSMUSG00000073155 | chr6:35202654-35213496 | chr6:34219246 - 35793923 | |
| **Slc17a4** | ENSMUSG00000021336 | chr13:23989607-24006876 | chr13:25090371 - 26931853 | |
| **Aimp2** | ENSMUSG00000029610 | chr5:144663573-144670716 | chr5:139008481 - 139739343 | |

| | | | | |
|---|---|---|---|---|
| **Fkbp4** | ENSMUSG00000030357 | chr6:128379753-128388695 | chr6:127554876 - 129517957 | |
| **Fermt2** | ENSMUSG00000037712 | chr14:46078467-46149793 | chr14:45679115 - 47174572 | |
| **Mup9** | ENSMUSG00000078686 | chr4:60430918-60434824 | chr4:59702851 - 62075481 | |
| **Stk40** | ENSMUSG00000042608 | chr4:125781201-125818273 | chr4:123718522 - 124472264 | |
| **Blvrb** | ENSMUSG00000040466 | chr7:28232997-28251163 | chr7:28218143 - 28450232 | |
| **Tnks1bp1** | ENSMUSG00000033955 | chr2:84888179-84913205 | chr2:126170268 - 127446389 | |
| **Pldn** | ENSMUSG00000005804 | chr2:122564239-122575211 | chr2:79562534 - 80052816 | |
| **Parp3** | ENSMUSG00000023249 | chr9:106372653-106379280 | chr9:108409204 - 108797772 | |
| **Mup11** | ENSMUSG00000073834 | chr4:60671338-60675283 | chr4:63068642 - 63718975 | |
| **Stt3a** | ENSMUSG00000032116 | chr9:36538860-36575264 | chr9:40177368 - 40327804 | |
| **0610005C13Rik** | ENSMUSG00000085214 | chr7:52823165-52830697 | chr7:48002471 - 50737476 | + |
| **Ccbl1** | ENSMUSG00000039648 | chr2:30040644-30061367 | chr2:28032120 - 30440952 | |
| **H2-T23** | ENSMUSG00000067212 | chr17:36166718-36169800 | chr17:35135941 - 37608019 | + |
| **Dcaf8** | ENSMUSG00000026554 | chr1:174078146-174126524 | chr1:170102936 - 170218291 | |
| **I7Rn6** | ENSMUSG00000062797 | chr7:97066039-97089709 | chr7:100211530 - 100340215 | |
| **Bbox1** | ENSMUSG00000041660 | chr2:110102854-110154717 | chr2:108029677 - 108172897 | |
| **C4a** | ENSMUSG00000015451 | chr17:34946037-34960409 | chr17:36883101 - 42313195 | |
| **Snhg3** | ENSMUSG00000085241 | chr4:131903929-131909601 | chr4:128174195 - 131589425 | |
| **4933403F05Rik** | ENSMUSG00000038121 | chr18:68419841-68459987 | chr18:67495307 - 68330850 | |
| **Uggt1** | ENSMUSG00000037470 | chr1:36198375-36301150 | chr1:33161004 - 33324614 | |
| **Tec** | ENSMUSG00000029217 | chr5:73146957-73259722 | chr5:74415579 - 75342154 | |
| **Ccndbp1** | ENSMUSG00000023572 | chr2:120834139-120842640 | chr2:124952741 - 133240610 | |
| **Rnf135** | ENSMUSG00000020707 | chr11:79997353-80013259 | chr11:77929454 - 81142274 | |
| **Mrpl45** | ENSMUSG00000018882 | chr11:97177030-97191234 | chr11:100723879 - 101189035 | |
| **Med16** | ENSMUSG00000013833 | chr10:79357452-79371683 | chr10:82162516 - 82279659 | |
| **Slc44a1** | ENSMUSG00000028412 | chr4:53453285-53635350 | chr4:53920065 - 54489084 | |
| **Ghr** | ENSMUSG00000055737 | chr15:3267760-3533492 | chr5:121667878 - 126901042 | |
| **Diablo** | ENSMUSG00000029433 | chr5:123959774-123974185 | chr15:6270762 - 6461965 | |
| **Zcrb1** | ENSMUSG00000022635 | chr15:93216528-93228765 | chr1:161391572 - 161990260 | |
| **Acbd6** | ENSMUSG00000033701 | chr1:157405250-157534363 | chr15:90396617 - 90833320 | |
| **Ube2j1** | ENSMUSG00000028277 | chr4:33118391-33139338 | chr4:32890927 - 33243086 | |
| **Ceacam1** | ENSMUSG00000074272 | chr7:26246721-26262644 | chr7:28450232 - 28693083 | + |
| **Abcg8** | ENSMUSG00000024254 | chr17:85075642-85099673 | chr17:85738579 - 86000112 | |
| **Bdh1** | ENSMUSG00000046598 | chr16:31422366-31458987 | chr16:29515137 - 29744925 | |
| **Man1a** | ENSMUSG00000003746 | chr10:53624594-53795602 | chr10:56694372 - 56846902 | |
| **Uroc1** | ENSMUSG00000034456 | chr6:90283283-90314545 | chr6:91305711 - 91473103 | |
| **Ext1** | ENSMUSG00000061731 | chr15:52895593-53177714 | chr15:55032882 - 55151291 | |
| **Rbms1** | ENSMUSG00000026970 | chr2:60588250-60801261 | chr2:56580607 - 56793825 | |
| **Gas2l1** | ENSMUSG00000034201 | chr11:4954135-4965330 | chr11:4752207 - 6298684 | |
| **Dnase2a** | ENSMUSG00000003812 | chr8:87432459-87446814 | chr8:83097136 - 83295171 | |
| **Rer1** | ENSMUSG00000029048 | chr4:154448219-154460491 | chr4:154269781 - 154567496 | |

| | | | | |
|---|---|---|---|---|
| **Ggnbp1** | ENSMUSG00000048731 | chr17:27110162-27173323 | chr17:21670293 - 22939394 | |
| **Zdhhc4** | ENSMUSG00000001844 | chr5:144078168-144090935 | chr5:148390455 - 148589118 | |
| **D2Wsu81e** | ENSMUSG00000039660 | chr2:30028967-30033979 | chr2:29305900 - 30440952 | |
| **Uqcrq** | ENSMUSG00000044894 | chr11:53241424-53244333 | chr10:81730570 - 81973250 | |
| **Mbd3** | ENSMUSG00000035478 | chr10:79855284-79862295 | chr11:50984651 - 51081191 | |
| **Sec11a** | ENSMUSG00000025724 | chr7:88049775-88092666 | chr7:84237109 - 84763098 | |
| **4930581F22Rik** | ENSMUSG00000070315 | chr9:34924313-34938507 | chr9:33781733 - 33881803 | |
| **Sigmar1** | ENSMUSG00000036078 | chr4:41685366-41703030 | chr4:45710215 - 46097584 | |
| **Nmd3** | ENSMUSG00000027787 | chr3:69525907-69552964 | chr3:70833134 - 71078481 | |
| **Dguok** | ENSMUSG00000014554 | chr6:83430211-83456963 | chr6:81487563 - 81699073 | |
| **Pfdn4** | ENSMUSG00000052033 | chr2:170321928-170344623 | chr2:165616921 - 165702733 | |
| **1810029B16Rik** | ENSMUSG00000025591 | chr8:68998229-69010429 | chr8:72763528 - 73988884 | |
| **Cfhr2** | ENSMUSG00000033898 | chr1:141701945-141915481 | chr11:69070924 - 69179181 | + |
| **Asgr1** | ENSMUSG00000020884 | chr11:69867587-69871396 | chr1:139521843 - 139731243 | |
| **Ubp1** | ENSMUSG00000009741 | chr9:113840052-113886317 | chr9:118200536 - 118322463 | |
| **Gapdh** | ENSMUSG00000057666 | chr6:125111871-125116485 | chr6:125623688 - 125808439 | |
| **Serpina11** | ENSMUSG00000063232 | chr12:105218453-105228167 | chr12:105256106 - 105294707 | |
| **Ndufc1** | ENSMUSG00000037152 | chr3:51208599-51212910 | chr3:49789152 - 50312406 | |
| **Mup16** | ENSMUSG00000078675 | chr4:61176624-61180563 | chr4:64434195 - 65164876 | + |
| **Apol9b** | ENSMUSG00000057346 | chr15:77559502-77566811 | chr15:80350701 - 80736040 | |
| **Apoe** | ENSMUSG00000002985 | chr7:20281458-20284515 | chr7:20107716 - 20245770 | |
| **Gusb** | ENSMUSG00000025534 | chr5:130464881-130478919 | chr5:129706169 - 130085213 | |
| **Bdh2** | ENSMUSG00000028167 | chr3:134944185-134967390 | chr3:137785490 - 138158838 | |
| **Bola3** | ENSMUSG00000045160 | chr6:83299141-83310130 | chr6:80550131 - 81145978 | |
| **Ftcd** | ENSMUSG00000001155 | chr10:76038393-76053083 | chr10:74913774 - 75004980 | |
| **Isoc2b** | ENSMUSG00000052605 | chr7:4796561-4817795 | chr7:5912336 - 6245609 | |
| **Plbd2** | ENSMUSG00000029598 | chr5:120933291-120953634 | chr5:113845542 - 116049346 | |
| **Cradd** | ENSMUSG00000045867 | chr10:94637380-94786767 | chr10:95892008 - 96014123 | |
| **BC017158** | ENSMUSG00000030780 | chr7:135414893-135441684 | chr7:132468721 - 133317628 | |
| **Fbxl20** | ENSMUSG00000020883 | chr11:97943868-98011717 | chr11:100891837 - 100977097 | |
| **Moap1** | ENSMUSG00000091931 | chr12:103978040-103996882 | chr12:103208606 - 103283852 | |
| **Mup10** | ENSMUSG00000078680 | chr4:60591132-60595071 | chr4:59981393 - 61881691 | |
| **Th1l** | ENSMUSG00000016253 | chr2:174241305-174253003 | chr2:178667105 - 178796335 | |
| **H2-Q6** | ENSMUSG00000073409 | chr17:35561795-35567000 | chr17:32322784 - 32433789 | |
| **Gnaq** | ENSMUSG00000024639 | chr19:16207321-16461953 | chr19:3250617 - 11384096 | |
| **Zap70** | ENSMUSG00000026117 | chr1:36818695-36839663 | chr2:74724352 - 74844038 | |
| **Metap1d** | ENSMUSG00000041921 | chr2:71291333-71363251 | chr1:33324614 - 33973089 | |
| **Gm2788** | ENSMUSG00000085995 | chr7:56133328-56141978 | chr7:60448010 - 60593267 | |
| **Zhx1** | ENSMUSG00000022361 | chr15:57878558-57908096 | chr15:56489504 - 56575711 | |
| **Adora1** | ENSMUSG00000042429 | chr1:136095800-136132008 | chr1:136588819 - 136870351 | |
| **Znhit1** | ENSMUSG00000059518 | chr5:137458034-137463891 | chr5:139739343 - 139827590 | |

| | | | | |
|---|---|---|---|---|
| **Clcc1** | ENSMUSG00000027884 | chr3:108456831-108481758 | chr3:108013537 - 108096986 | |
| **Rmnd1** | ENSMUSG00000019763 | chr10:5914153-5944626 | chr10:3082438 - 3217673 | + |
| **Fxyd1** | ENSMUSG00000036570 | chr7:31836700-31840675 | chr7:35979575 - 35926459 | |

**Table S5. The connectivity of drugs to eQTLs through drug disposition enzymes.** For each drug (column 1), reported are the relevant drug disposition processes (column 2). For each such process, reported are the eQTL-associated genes that are involved in the process, either based on a direct evidence regarding the gene (column 3), or an evidence regarding the EC number of a gene (column 4).

| Drug | Drug disposition process | Enzymes (direct evidence) | Enzymes (indirect evidence) |
|---|---|---|---|
| 5-fluorouracil | formation of 5-fluorouracil | Cyp3a16 | Cyp2c40,Cyp3a13,Cyp2c44,Cyp2d12,Cyp2d11,Cyp3a25 |
| 6-mercaptopurine | metabolism of 6-mercaptopurine | Tpmt | - |
| Acetaminophen | toxicity of acetaminophen | Nr1i3 | - |
| Acetaminophen | glucuronidation of acetaminophen | Ugt1a6b | - |
| Acetaminophen | activation of acetaminophen | Cyp3a16 | Cyp2c40,Cyp3a13,Cyp2c44,Cyp2d12,Cyp2d11,Cyp3a25 |
| Acetaminophen | metabolism of acetaminophen | - | Cyp2c40,Cyp3a13,Cyp3a16,Cyp2c44,Cyp2d12,Cyp2d11,Cyp3a25 |
| Aldesleukin | synthesis of aldesleukin | Cd1d2 | - |
| Alitretinoin | metabolism of alitretinoin | Aldh8a1,Rdh9 | Cyp2c40,Cyp3a13,Cyp3a16,Cyp2c44,Cyp2d12,Cyp2d11,Cyp3a25 |
| Almotriptan | metabolism of almotriptan | Fmo1 | Cyp2c40,Cyp3a13,Cyp3a16,Cyp2c44,Cyp2d12,Cyp2d11,Cyp3a25 |
| Alprazolam | metabolism of alprazolam | Cyp3a16 | Cyp2c40,Cyp3a13,Cyp2c44,Cyp2d12,Cyp2d11,Cyp3a25 |
| Amiodarone | metabolism of amiodarone | Cyp3a16 | Cyp2c40,Cyp3a13,Cyp2c44,Cyp2d12,Cyp2d11,Cyp3a25 |
| Atorvastatin | transport of atorvastatin | Slco1a1 | - |
| Bosentan | uptake of bosentan | Slco1a1 | - |
| Caffeine | metabolism of caffeine | - | Cyp2c40,Cyp3a13,Cyp3a16,Cyp2c44,Cyp2d12,Cyp2d11,Cyp3a25 |
| Cerivastatin | metabolism of cerivastatin | - | Cyp2c40,Cyp3a13,Cyp3a16,Cyp2c44,Cyp2d12,Cyp2d11,Cyp3a25 |
| Chloramphenicol | glucuronidation of chloramphenicol | - | Ugt1a10,Ugt1a6b,Ugt1a6a,Ugt3a1,Ugt3a2 |
| Cimetidine | uptake of cimetidine | Slco1a1 | - |
| Cladribine | phosphorylation of cladribine | Dguok | - |
| Cocaine | metabolism of cocaine | - | Ces3a,Ces2h,Ces1g |
| Coumarin | metabolism of coumarin | Ugt1a6b,Ugt1a10 | Ugt1a6a,Ugt3a1,Ugt3a2,Cyp2c40,Cyp3a13,Cyp3a16,Cyp2c44,Cyp2d12,Cyp2d11,Cyp3a25 |
| Cyclophosphamide | metabolism of cyclophosphamide | - | Cyp2c40,Cyp3a13,Cyp3a16,Cyp2c44,Cyp2d12,Cyp2d11,Cyp3a25 |
| Desipramine | conversion of desipramine | - | Cyp2c40,Cyp3a13,Cyp3a16,Cyp2c44,Cyp2d12,Cyp2d11,Cyp3a25 |
| Dextromethorphan | modification of dextromethorphan | Cyp3a16 | Cyp2c40,Cyp3a13,Cyp2c44,Cyp2d12,Cyp2d11,Cyp3a25 |
| Diazepam | metabolism of diazepam | Cyp3a16 | Cyp2c40,Cyp3a13,Cyp2c44,Cyp2d12,Cyp2d11,Cyp3a25 |

| | | | |
|---|---|---|---|
| Diclofenac | metabolism of diclofenac | - | Ugt1a10,Ugt1a6b,Ugt1a6a,Ugt3a1,Ugt3a2,Cyp2c40,Cyp3a13,Cyp3a16,Cyp2c44,Cyp2d12,Cyp2d11,Cyp3a25 |
| Diflunisal | glucuronidation of diflunisal | - | Ugt1a10,Ugt1a6b,Ugt1a6a,Ugt3a1,Ugt3a2 |
| D-methylphenidate | hydrolysis of D-methylphenidate | - | Ces3a,Ces2h,Ces1g |
| Docetaxel | metabolism of docetaxel | Cyp3a16 | Cyp2c40,Cyp3a13,Cyp2c44,Cyp2d12,Cyp2d11,Cyp3a25 |
| Epoprostenol | synthesis of epoprostenol | Tnfsf10 | - |
| Etoposide | metabolism of etoposide | Cyp3a16 | Cyp2c40,Cyp3a13,Cyp2c44,Cyp2d12,Cyp2d11,Cyp3a25 |
| Fluoxetine | modification of fluoxetine | - | Cyp2c40,Cyp3a13,Cyp3a16,Cyp2c44,Cyp2d12,Cyp2d11,Cyp3a25 |
| Flutamide | metabolism of flutamide | - | Cyp2c40,Cyp3a13,Cyp3a16,Cyp2c44,Cyp2d12,Cyp2d11,Cyp3a25 |
| Haloperidol | binding of haloperidol | Sigmar1 | - |
| Haloperidol | metabolism of haloperidol | Cyp3a16 | Cyp2c40,Cyp3a13,Cyp2c44,Cyp2d12,Cyp2d11,Cyp3a25 |
| Heparin | synthesis of heparin | Ext1 | - |
| Heparin | binding of heparin | Fgf1,Sulf2 | - |
| Hydrocortisone | metabolism of hydrocortisone | Cyp3a16 | Cyp2c40,Cyp3a13,Cyp2c44,Cyp2d12,Cyp2d11,Cyp3a25 |
| Ibuprofen | metabolism of ibuprofen | - | Cyp2c40,Cyp3a13,Cyp3a16,Cyp2c44,Cyp2d12,Cyp2d11,Cyp3a25 |
| Ifosfamide | metabolic process of ifosfamide | - | Cyp2c40,Cyp3a13,Cyp3a16,Cyp2c44,Cyp2d12,Cyp2d11,Cyp3a25 |
| Imipramine | metabolism of imipramine | - | Cyp2c40,Cyp3a13,Cyp3a16,Cyp2c44,Cyp2d12,Cyp2d11,Cyp3a25 |
| Indinavir | metabolism of indinavir | Cyp3a16 | Cyp2c40,Cyp3a13,Cyp2c44,Cyp2d12,Cyp2d11,Cyp3a25 |
| Indomethacin | glucuronidation of indomethacin | - | Ugt1a10,Ugt1a6b,Ugt1a6a,Ugt3a1,Ugt3a2 |
| Irinotecan | hydrolysis of irinotecan | Ces2h,Ces3a | Ces1g |
| **Irinotecan** | **metabolism of irinotecan** | Gusb,Ces2h,Ces3a,Ugt1a10,Ugt1a6b,Ugt1a6a | Ces1g,Ugt3a1,Ugt3a2 |
| Lovastatin | transport of lovastatin | Slco1a1 | - |
| Methadone | metabolism of methadone | - | Cyp2c40,Cyp3a13,Cyp3a16,Cyp2c44,Cyp2d12,Cyp2d11,Cyp3a25 |
| Midazolam | metabolism of midazolam | Cyp3a16 | Cyp2c40,Cyp3a13,Cyp2c44,Cyp2d12,Cyp2d11,Cyp3a25 |
| Morphine | glucuronidation of morphine | Ugt1a6b,Ugt1a10 | Ugt1a6a,Ugt3a1,Ugt3a2,Cyp2c40,Cyp3a13,Cyp3a16,Cyp2c44,Cyp2d12,Cyp2d11,Cyp3a25 |
| Morphine | conversion of morphine | Ugt1a10 | Ugt1a6b,Ugt1a6a,Ugt3a1,Ugt3a2 |
| Muscimol | binding of muscimol | Adhfe1 | - |
| Nicotine | metabolism of nicotine | Ugt1a10,Ugt1a6b,Ugt1a6a,Fmo1,Cyp2c40,Cyp3a13,Cyp3a16,Cyp2c44 | Ugt3a1,Ugt3a2,Cyp2d12,Cyp2d11,Cyp3a25 |
| Nifedipine | metabolism of nifedipine | Cyp3a16 | Cyp2c40,Cyp3a13,Cyp2c44,Cyp2d12,Cyp2d11,Cyp3a25 |

| | | | |
|---|---|---|---|
| Norepinephrine | flow of norepinephrine | Adora1 | - |
| Norepinephrine | release of norepinephrine | Adora1,Nr2f6 | - |
| Norepinephrine | concentration of norepinephrine | App | - |
| Omeprazole | metabolism of omeprazole | - | Cyp2c40,Cyp3a13,Cyp3a16,Cyp2c44,Cyp2d12,Cyp2d11,Cyp3a25 |
| Paclitaxel | uptake of paclitaxel | Slco1a1 | - |
| Paclitaxel | metabolism of paclitaxel | Cyp3a16 | Cyp2c40,Cyp3a13,Cyp2c44,Cyp2d12,Cyp2d11,Cyp3a25 |
| Pravastatin | transport of pravastatin | Slco1a1 | - |
| Propranolol | glucuronidation of propranolol | - | Ugt1a10,Ugt1a6b,Ugt1a6a,Ugt3a1,Ugt3a2 |
| Ritonavir | metabolism of ritonavir | Cyp3a16 | Cyp2c40,Cyp3a13,Cyp2c44,Cyp2d12,Cyp2d11,Cyp3a25 |
| Simvastatin | transport of simvastatin | Slco1a1 | - |
| Tamoxifen | metabolism of tamoxifen | Cyp3a16 | Cyp2c40,Cyp3a13,Cyp2c44,Cyp2d12,Cyp2d11,Cyp3a25 |
| Terfenadine | metabolism of terfenadine | Cyp3a16 | Cyp2c40,Cyp3a13,Cyp2c44,Cyp2d12,Cyp2d11,Cyp3a25 |
| Thalidomide | metabolism of thalidomide | Cyp3a16 | Cyp2c40,Cyp3a13,Cyp2c44,Cyp2d12,Cyp2d11,Cyp3a25 |
| Theophylline | metabolism of theophylline | Cyp3a16 | Cyp2c40,Cyp3a13,Cyp2c44,Cyp2d12,Cyp2d11,Cyp3a25 |
| Thioguanine | metabolism of thioguanine | Tpmt | - |
| Tolbutamide | metabolism of tolbutamide | Cyp3a16 | Cyp2c40,Cyp3a13,Cyp2c44,Cyp2d12,Cyp2d11,Cyp3a25 |
| Tretinoin | synthesis of tretinoin | Rdh9 | - |
| Tretinoin | metabolism of tretinoin | Aldh8a1,Cyp3a16,Cyp3a13,Rdh9 | Cyp2c40,Cyp2c44,Cyp2d12,Cyp2d11,Cyp3a25 |
| Tretinoin | hydroxylation of tretinoin | - | Cyp2c40,Cyp3a13,Cyp3a16,Cyp2c44,Cyp2d12,Cyp2d11,Cyp3a25 |
| Tretinoin | glucuronidation of tretinoin | - | Ugt1a10,Ugt1a6b,Ugt1a6a,Ugt3a1,Ugt3a2 |
| Triazolam | metabolism of triazolam | Cyp3a16 | Cyp2c40,Cyp3a13,Cyp2c44,Cyp2d12,Cyp2d11,Cyp3a25 |
| **Trichloroethylene (TCE)** | **metabolism of TCE** | Cyp2c40,Cyp2c44,Gsta2,Gstm6,Mgst3,Ccbl1, Ccbl2 | Cyp3a13,Cyp3a16,Cyp2d12 |
| Verapamil | metabolism of verapamil | Cyp3a16 | Cyp2c40,Cyp3a13,Cyp2c44,Cyp2d12,Cyp2d11,Cyp3a25 |
| Voriconazole | metabolism of voriconazole | Cyp3a16 | Cyp2c40,Cyp3a13,Cyp2c44,Cyp2d12,Cyp2d11,Cyp3a25 |

**Table S6. A summary of the eQTL-drug map**. For each drug (column 1), the table presents the relevant eQTL-associated drug disposition genes (columns 2,3) and the number of underlying eQTLs (column 4). The annotation of enzymes is either based on direct evidence on particular enzymes (column 2) or based on information about EC numbers (column 3). The total number of different enzyme classes is indicated in column 5 (including both direct and indirect evidence). Abbreviations: CYPs, Cytochromes P450; CES, Carboxyl esterase; UGTs, UDP-glucuronosyl transferases; GSTs, Glutathione S-transferases (see **Table 1** for details).

| Drug | Enzymes (direct) | Enzymes (indirect) | # eQTLs | # Enzyme classes |
|---|---|---|---|---|
| Tretinoin | Aldh8a1,CYPs,Rdh9 | UGTs | >2 | 4 |
| Acetaminophen | Nr1i3, UGTs, CYPs | - | >2 | 3 |
| Alitretinoin | Aldh8a1,Rdh9 | CYPs | >2 | 3 |
| Heparin | Fgf1,Sulf2,Ext1 | - | >2 | 3 |
| Irinotecan | Gusb,CES,UGTs | - | >2 | 3 |
| Nicotine | UGTs,Fmo1,CYPs | - | >2 | 3 |
| Norepinephrine | Adora1,Nr2f6,App | - | >2 | 3 |
| Trichloroethylene (TCE) | CYPs,GSTs,Ccbl1/2 | - | >2 | 3 |
| Almotriptan | Fmo1 | CYPs | >2 | 2 |
| Coumarin | UGTs | CYPs | >2 | 2 |
| Diclofenac | - | UGTs, CYPs | >2 | 2 |
| Haloperidol | Sigmar1,CYPs | - | >2 | 2 |
| Paclitaxel | Slco1a1,CYPs | - | >2 | 2 |
| Caffeine | - | CYPs | >2 | 1 |
| Cerivastatin | - | CYPs | >2 | 1 |
| Cyclophosphamide | - | CYPs | >2 | 1 |
| Desipramine | - | CYPs | >2 | 1 |
| Fluoxetine | - | CYPs | >2 | 1 |
| Flutamide | - | CYPs | >2 | 1 |
| Ibuprofen | - | CYPs | >2 | 1 |
| Ifosfamide | - | CYPs | >2 | 1 |
| Imipramine | - | CYPs | >2 | 1 |
| Methadone | - | CYPs | >2 | 1 |
| Omeprazole | - | CYPs | >2 | 1 |
| 5-fluorouracil | CYPs | - | >2 | 1 |
| Alprazolam | CYPs | - | >2 | 1 |
| Amiodarone | CYPs | - | >2 | 1 |
| Dextromethorphan | CYPs | - | >2 | 1 |
| Diazepam | CYPs | - | >2 | 1 |
| Docetaxel | CYPs | - | >2 | 1 |
| Etoposide | CYPs | - | >2 | 1 |
| Hydrocortisone | CYPs | - | >2 | 1 |
| Indinavir | CYPs | - | >2 | 1 |

| | | | | |
|---|---|---|---|---|
| Midazolam | CYPs | - | >2 | 1 |
| Nifedipine | CYPs | - | >2 | 1 |
| Ritonavir | CYPs | - | >2 | 1 |
| Tamoxifen | CYPs | - | >2 | 1 |
| Terfenadine | CYPs | - | >2 | 1 |
| Thalidomide | CYPs | - | >2 | 1 |
| Theophylline | CYPs | - | >2 | 1 |
| Tolbutamide | CYPs | - | >2 | 1 |
| Triazolam | CYPs | - | >2 | 1 |
| Verapamil | CYPs | - | >2 | 1 |
| Voriconazole | CYPs | - | >2 | 1 |
| Morphine | UGTs | - | 2 | 1 |
| Chloramphenicol | - | UGTs | 2 | 1 |
| Diflunisal | - | UGTs | 2 | 1 |
| Indomethacin | - | UGTs | 2 | 1 |
| Propranolol | - | UGTs | 2 | 1 |
| 6-mercaptopurine | Tpmt | - | 1 | 1 |
| Aldesleukin | Cd1d2 | - | 1 | 1 |
| Atorvastatin | Slco1a1 | - | 1 | 1 |
| Bosentan | Slco1a1 | - | 1 | 1 |
| Cimetidine | Slco1a1 | - | 1 | 1 |
| Cladribine | Dguok | - | 1 | 1 |
| Epoprostenol | Tnfsf10 | - | 1 | 1 |
| Lovastatin | Slco1a1 | - | 1 | 1 |
| Muscimol | Adhfe1 | - | 1 | 1 |
| Pravastatin | Slco1a1 | - | 1 | 1 |
| Simvastatin | Slco1a1 | - | 1 | 1 |
| Thioguanine | Tpmt | - | 1 | 1 |
| Cocaine | - | CES | 1 | 1 |
| D-methylphenidate | - | CES | 1 | 1 |
